# Targeting *Streptococcus pyogenes* atpF protein for multi-epitope vaccine development: a genomics-driven immunoinformatics strategy

**DOI:** 10.1016/j.jgeb.2025.100546

**Published:** 2025-08-05

**Authors:** Manisha Agarwal, Sanjeeb Handique, Sanchaita Rajkhowa, Abhichandan Das, Debashis Panda, Sami A. Al-Hussain, Magdi E.A. Zaki

**Affiliations:** a Centre for Biotechnology and Bioinformatics, Dibrugarh University, Dibrugarh 786004 Assam, India; b Bioinformatics and Computational Biology Centre, DBT-APSCS&T CoE for Bioresources and Sustainable Development, Kimin 791121 Arunachal Pradesh, India; c DBT-APSCS&T CoE for Bioresources and Sustainable Development, Kimin 791121 Arunachal Pradesh, India; d Department of Chemistry, Imam Mohammad Ibn Saud Islamic University (IMSIU), Riyadh, Saudi Arabia

**Keywords:** *Streptococcus pyogenes*, Multi-epitope vaccine, Reverse vaccinology, ATP synthase F1 subunit

## Abstract

*Streptococcus pyogenes*, a medium-priority pathogen on the WHO’s 2024 Bacterial Pathogen Priority List, is a major cause of infectious disease-related mortality. The increasing prevalence of antibiotic resistance, coupled with the absence of a licensed vaccine due to the pathogen’s genetic diversity and autoimmune concerns, underscores the need for novel therapeutic strategies. This study employs reverse vaccinology and subtractive proteomics to design a multi-epitope vaccine targeting atpF, a conserved extracellular protein essential for ATP synthesis. The atpF protein was identified based on its high antigenicity and functional importance in *S. pyogenes*. Three vaccine constructs (SM1, SM2, and SM3) were designed by integrating antigenic B-cell and T-cell epitopes with immune-modulating adjuvants and linkers. Physicochemical and immunological assessments confirmed their stability, solubility, and antigenicity. Molecular modeling identified SM1 as the most promising candidate, demonstrating superior immune receptor binding affinity and flexible epitope interactions, facilitating effective antibody recognition. *In silico* immune simulations further demonstrated SM1′s potential to elicit strong humoral and cellular immune responses, while codon optimization confirmed efficient expression in *E. coli*. These findings introduce atpF as a promising vaccine target and highlight SM1′s potential as a viable vaccine candidate. However, experimental validation is essential to confirm its efficacy, safety, and immunogenicity *in vivo*. This study underscores the role of computational modeling in accelerating vaccine development, providing a strategic alternative to traditional approaches.

## Highlights

• *Streptococcus pyogenes* causes severe infections with limited treatment options.
• Subunit vaccines offer safe, effective, and affordable alternatives to traditional ones.
• Reverse vaccinology uncovered novel antigenic targets in *S. pyogenes*.
• Three vaccine candidates were designed; SM1 showed strongest immune interaction.
• SM1 is predicted to induce robust, broad immune responses across populations.

## Graphical abstract

**Figure:**
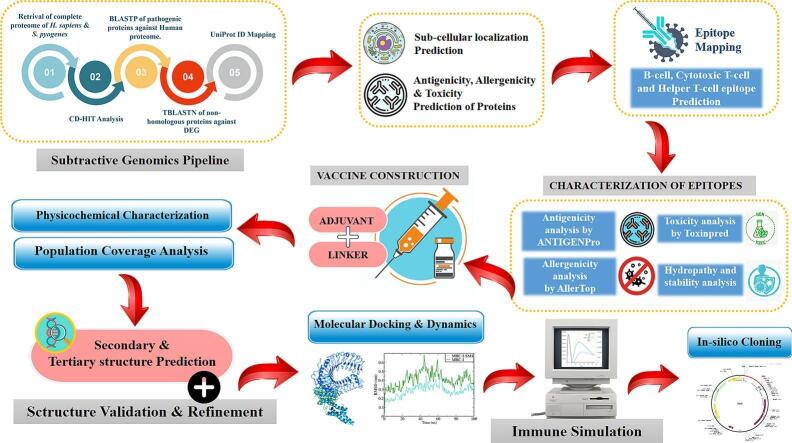

## 1 Introduction

*Streptococcus pyogenes* (Group A *Streptococcus*, GAS) is a gram-positive bacterium that thrives in the human host, typically colonizing the upper respiratory tract and skin. It is responsible for a wide spectrum of human diseases, ranging from mild conditions such as pharyngitis and impetigo to severe, life-threatening illnesses like streptococcal toxic shock syndrome (STSS), septicemia, and necrotizing fasciitis. Recurrent infections can result in autoimmune complications, notably rheumatic fever, which may progress to rheumatic heart disease (RHD), a leading cause of morbidity and mortality associated with GAS.^1^ Globally, RHD affects approximately 15.6 million people and accounts for over 233,000 deaths annually, with the highest prevalence observed in children aged 5 to 14 in sub-Saharan Africa, followed by populations in Australia, New Zealand, and Asia.^2^ In India alone, RHD prevalence ranges from 0.12 to 4.54 per 1000 children, translating to 0.44 to 3.37 million cases.^3^ GAS infections disproportionately burden low- and middle-income countries and marginalized populations, including Pacific Islanders, Indigenous Australians, and Native American/Alaskan Native communities. In addition to autoimmune complications, GAS infections impose a substantial global health and economic burden, with over 616 million cases of pharyngitis and 111 million cases of GAS-associated skin infections, such as pyoderma, reported annually.^4^ The socioeconomic impact of these infections is amplified in regions with limited healthcare resources, where delayed or inadequate treatment exacerbates disease progression.

GAS exhibits a remarkable capacity for invading a wide range of host cell types, including endothelial cells, epithelial cells, and professional phagocytes. This pathogen's evasion strategies allow it to circumvent the host's innate immune defenses. Specifically, GAS can avoid being engulfed and destroyed by phagocytes and can interfere with selective autophagy. Additionally, GAS can evade light chain 3-associated phagocytosis (LAP), a specialized form of autophagy that deals with extracellular pathogens. Furthermore, GAS can modulate host inflammatory responses, thereby promoting its own survival and persistence within the host. These evasion mechanisms collectively enable GAS to establish infections and contribute to its pathogenicity.^5^

GAS exhibits significant genetic diversity, characterized by a broad array of virulence factors that interact with various host defenses. Among these virulence factors, the M protein and streptococcal pyrogenic exotoxins are particularly prominent in the pathogenesis of GAS.^6^ The M protein, a major virulence factor encoded by the emm gene, is crucial to the pathogenesis and immune evasion mechanisms of GAS.^7^ This protein serves as a key immunological determinant, and its molecular typing remains the primary method for characterizing the global genetic diversity of GAS.^8^ To date, over 250 distinct emm types have been identified and cataloged, reflecting the extensive genetic variability within the M protein.^9^ This diversity poses significant challenges to vaccine development, particularly for M protein-specific vaccines. Variations in the amino acid sequence of the N-terminal region, the most antigenically variable segment of the M protein, drive the emergence of distinct GAS serotypes.^10^ These genetic differences produce immunologically unique surface structures, enabling novel serotypes to evade host antibodies generated during previous infections.^11^ This immune evasion mechanism complicates the development of broadly protective vaccines, as antibodies targeting one serotype often fail to provide cross-protection against others.^12^

GAS also expresses a variety of surface adhesins, including pili (Spy0130, Spy0128, Cpa), collagen-like proteins (Scl1, Scl2), fibronectin-binding proteins (PrtF1, PrtF2, SfbI, SfbII, SOF, Fbaa, Fbab), laminin-binding proteins (Lbp, Shr), and plasminogen-binding proteins (GAPDH, SEN). These adhesins facilitate the attachment and invasion of host tissues. Furthermore, GAS secretes a variety of factors that contribute to its virulence. Streptokinase (Ska) interacts with the complement system, promoting fibrinolysis and modulating neutrophil activity. Streptolysin S (SLS) is another critical virulence factor that promotes the lysis of host cells and aids in immune evasion. Additionally, GAS produces hyaluronidase and cysteine proteinases, which degrade host tissues and contribute to the pathogen’s invasive capabilities.^1^ These secreted factors collectively enhance the pathogenic potential of GAS, enabling it to effectively colonize and cause disease in the host.

Despite the substantial global health burden imposed by *Streptococcus pyogenes*, the development and licensure of vaccines against this pathogen remain elusive. In the absence of a commercially available vaccine for GAS, clinical management predominantly depends on the use of antibiotics for the treatment or prevention of infection. Antibiotics are employed to target and eliminate GAS bacteria, thereby controlling the spread of the infection and reducing associated morbidity and mortality. While GAS typically exhibits sensitivity to β-lactam antibiotics universally, the development of resistance mechanisms against alternative treatment options like macrolides and lincosamides is a common occurrence. Such resistance often leads to recurring infections, treatment ineffectiveness, and adverse patient outcomes.^13^, ^14^, ^15^ Moreover, the continual emergence of subclinical β-lactam resistance in GAS remains a significant challenge in clinical settings.^16^, ^17^ The 2024 Bacterial Pathogen Priority List (BPPL), published by the World Health Organization (WHO), highlights *Streptococcus pyogenes* as a pathogen of substantial global concern. This bacterium is classified as a medium-priority pathogen, based on WHO criteria that evaluate factors such as moderate challenges in treatment, a notable disease burden characterized by mortality and morbidity, and emerging trends in antibiotic resistance. Pathogens within this priority group often pose distinct challenges related to their preventability or transmissibility and, while not uniformly critical, may represent significant threats to specific populations or regions. This classification underscores the pressing need for the development of an effective vaccine to combat *S. pyogenes*.^18^

The pursuit of a vaccine against scarlet fever commenced over two centuries ago, predating the definitive identification of its causative agent.^19^, ^20^ However, progress towards developing a Group A *Streptococcus* vaccine was impeded by the recognition, since the 1930s, of cross-reactivity between antibodies produced against certain streptococcal components and human tissues. Additionally, safety concerns emerged in the 1960s regarding the potential for these vaccines to exacerbate the development of ARF and subsequent cardiac complications.^21^ These scientific challenges are exacerbated by historical regulatory and commercial barriers that have impeded the development of GAS vaccines.^22^ A significant obstacle was the 25-year ban imposed by the U.S. Food and Drug Administration (FDA) due to concerns about the autoimmune risks associated with GAS antigens. Despite the lifting of this ban in 2005, advancements in GAS vaccine development have been limited, with only four vaccine candidates progressing to early-stage human trials since then.^23^ Despite the shortcomings of prior efforts, ongoing research is dedicated to the development of more effective vaccines against *Streptococcus pyogenes*.

The traditional methodologies for drug and vaccine development are often constrained by high costs, extended timelines, and the identification of a limited number of therapeutic targets. In contrast, computational modeling has emerged as a transformative approach, leveraging OMICS data to identify drug and vaccine targets with greater precision and efficiency, thereby reducing the attrition rates of candidates in clinical trials.^24^ The integration of big data analytics has further advanced this field, enabling the discovery of novel therapeutic targets through sophisticated computational techniques. One such approach, subtractive genomics, exemplifies the potential of computational modeling as a comprehensive alternative to empirical testing. This method involves a systematic analysis of pathogen and host proteomes to identify pathogen-specific proteins with therapeutic potential, while excluding host-homologous proteins to minimize off-target effects.^25^ Subtractive genomics has proven effective in identifying unique, pathogen-specific targets, providing a robust foundation for vaccine and drug design.^26^

The incorporation of immunoinformatics and reverse vaccinology has further revolutionized computer-aided vaccine design. Reverse vaccinology allows for the identification of vaccine candidates and therapeutic targets, even for pathogens that are challenging to culture *in vitro*.^27^, ^28^ Epitope-based vaccine design, a key outcome of these computational advancements, offers distinct advantages over conventional vaccines. Unlike traditional approaches, which rely heavily on *in vitro* culture models and are associated with prolonged development timelines, epitope-based vaccine design utilizes computational tools to rapidly identify and target immunogenic protein regions across bacterial and viral proteomes. These vaccines are not only precise and cost-effective to formulate but also elicit targeted immune responses without the risk of adverse cytokine storms.^29^

In recent years, computational modeling has gained widespread application in the design of epitope-based vaccines targeting various human and animal pathogens, with several candidates demonstrating successful validation in animal studies.^30^, ^31^ This underscores the potential of *in silico* exploration as a foundational step in vaccine development. By enabling the identification of immunodominant epitope regions within vaccine candidate antigens, computational approaches provide invaluable insights, paving the way for advancements in vaccinology and the development of next-generation vaccines.^32^

This study employs a reverse vaccinology approach to design a multi-epitope subunit vaccine targeting the atpF protein of *Streptococcus pyogenes*, emphasizing its novelty as a vaccine target. The atpF protein is a critical component of the ATP synthase complex, which is indispensable for bacterial energy metabolism. It facilitates ATP synthesis from ADP and inorganic phosphate by utilizing the proton gradient across the bacterial membrane. Distinct from conventional vaccine targets, the atpF protein plays a central role in maintaining the functionality of the ATP synthase complex and is vital for cellular energy production. ATP, as the primary energy currency, drives essential metabolic pathways and cellular processes, ensuring the survival, adaptability, and virulence of *S. pyogenes*. The pathogen’s dependence on ATP synthesis allows it to persist in diverse host environments, evade immune defenses, and exhibit resilience against antibiotic treatments.^33^ This underscores the atpF protein’s significance as an essential component for bacterial viability and a promising, yet underexplored, target for vaccine development.

By focusing on the atpF protein, this study introduces a novel strategy to combat *S. pyogenes* infections, addressing the critical need for innovative and effective vaccine candidates. Targeting a protein so deeply integrated into the pathogen’s metabolic and survival machinery offers a robust framework for developing prophylactic solutions against this prominent human pathogen.

## 2 Materials and methods

### 2.1 Subtractive genomics approach

Subtractive genomics is an effective technique used to distinguish between the proteomes and metabolic pathways of hosts and pathogens by identifying proteins crucial for the microorganism that are absent in the host.^34^ This approach is instrumental in discovering unique drug and vaccine targets vital for pathogen survival while avoiding disruption of the host’s metabolic functions.^35^ In this study, proteomic data for *Homo sapiens* and *Streptococcus pyogenes* were retrieved from NCBI (ftp://ftp.ncbi.nlm.nih.gov/genomes/), and the DEG database was accessed from https://tubic.tju.edu.cn/deg/ to assess the essentiality of potential vaccine targets.

The entire proteome of *S. pyogenes* was filtered at a 60% identity threshold using the CD- HIT tool.^36^ Proteins exhibiting sequence identity above 60% were considered paralogous, and such duplicate sequences were excluded, retaining only non-paralogous sequences for further analysis. The non-paralogous sequences were analyzed using BLASTp against the *Homo sapiens* genome, applying an E-value threshold of 10^−5^ to identify significant matches. To further refine the dataset, sequences showing duplication were filtered out at a 29% identity threshold. Non-homologous sequences were subsequently identified using a custom Python script, ensuring a highly specific and unique dataset.

Essential proteins, defined as those playing critical roles in cellular metabolism, were identified by performing TBLASTN of the non-homologous *S. pyogenes* proteins against the DEG. Given that TBLASTN queries protein sequences against a nucleotide database, it translates nucleotide sequences into all six possible reading frames (three per strand), enabling a comprehensive comparison. Proteins were classified as essential based on a stringent E-value threshold of 10^−100^ and a minimum score cut-off of 100, ensuring high specificity. To enhance dataset quality, sequences with more than 93% identity were removed to eliminate redundancy. This rigorous filtering process resulted in a refined set of proteins that were both non-homologous to the host and critical for the survival of *S. pyogenes*. The resultant nucleotide sequences were translated into protein sequences using an in-house Perl script. Subsequently, protein accession numbers from the resultant dataset were converted to gene names via the UniProt ID mapping tool. This process yielded a robust set of potential drug targets for further investigation.

### 2.2 Determination of subcellular localization

The subcellular localization of proteins is pivotal in vaccine design, offering critical insights into their functional roles within the cellular milieu. To analyze the subcellular localization of essential and non-homologous proteins, we utilized the BUSCA web server, a benchmarked and reliable tool for the characterization of query protein sequences. BUSCA integrates multiple advanced prediction tools, including DeepSig, TPPred3, PredGPI, BetAware, and ENSEMBLE3.0, as well as localization-specific predictors like MemLoci, BaCelLo, and SChloro. This platform was selected for its ability to centralize diverse resources, offering not only precise localization predictions but also detailed annotation of critical features such as signal peptides, transit peptides, GPI anchors, and transmembrane domains.^37^ The principle behind subcellular localization involves conducting BLAST searches of non-homologous proteins against a database of proteins with known subcellular locations. This allows for the classification of proteins based on their cellular compartments. Using this approach, proteins were categorized into distinct types according to their cellular locations.^38^

Surface proteins on cells are ideal targets for vaccine development due to their immune system accessibility and the presence of multiple epitopes recognized by B and T cells.^39^ B cells generate antibodies for humoral immunity, while T cells drive cellular immunity to eliminate infected cells. This dual activation promotes a comprehensive immune response, offering both immediate and sustained protection. Consequently, the focus on surface proteins in vaccine design is a strategic choice, prioritizing the most immunogenic and accessible targets to enhance vaccine efficacy.^40^

### 2.3 Antigenic, allergenic and toxicity prediction of surface proteins

Identifying allergens, toxicity, and antigenicity is crucial for developing vaccines that are safe, effective, and broadly acceptable, ensuring they provoke the desired immune response while minimizing adverse reactions, thereby enhancing public confidence and meeting regulatory standards.

Antigenicity plays a pivotal role in inducing a protective immune response, enabling the immune system to identify and retain memory of the pathogen, thus ensuring long-term immunity. ANTIGENpro, a sequence-based prediction tool, was utilized to identify highly antigenic proteins for vaccine development, ensuring optimal efficacy. This tool, trained on a comprehensive, non-redundant dataset derived primarily from protein microarray analyses, predicts the likelihood of a protein being a protective antigen. It demonstrates an accuracy of 82% in classifying known protective antigens using protein microarray datasets and achieves 76% accuracy on a combined dataset through cross-validation. Importantly, ANTIGENpro minimizes false positives by effectively distinguishing antigenic regions from non-antigenic sequences, thereby enhancing prediction precision.^41^ The antigenic probability is expressed as a score ranging from 0 to 1, with higher scores indicating stronger antigenicity.^42^ For this study, a threshold score of 0.5 was selected to maximize sensitivity and specificity, ensuring the identification of the most antigenic surface proteins while maintaining reliability in the predictions.

Accurate allergen detection is critical for preventing adverse immune responses, ranging from mild skin rashes to life-threatening anaphylaxis. To evaluate the allergenic potential of antigenic proteins, the AllerTOP v2.0 server was utilized with a threshold value of 0.4, ensuring that selected candidates are unlikely to trigger allergic reactions, thereby enhancing the safety profile of vaccine epitopes.^43^ Eliminating toxicity is fundamental for vaccine safety, as toxic components can lead to harmful side effects and long-term health issues. To address this, ToxinPred was employed to evaluate the toxicity of epitopes. Non-toxic epitopes were selected for further analysis based on a threshold of 0.5, achieving an accuracy range of 70–80%.^44^

### 2.4 B-cell epitopes selection

B cells play a crucial role in vaccine development by recognizing antigens through their B cell receptors (BCRs). This recognition triggers B cell activation, followed by clonal expansion and differentiation into antibody-secreting plasma cells. To identify potential target antigens, linear B-cell epitopes were predicted using the ABCpred server and the Immune Epitope Database (IEDB).^44^, ^45^

ABCpred was chosen for B-cell epitope prediction due to its extensive benchmarking and robust design based on feed-forward (FNN) and recurrent neural networks (RNN). When evaluated through five fold cross-validation, ABCpred demonstrated an overall prediction accuracy of 65.93%, with sensitivity, specificity, and positive predictive values of 67.14%, 64.71%, and 65.61%, respectively. These metrics highlight its balanced performance, making it a suitable choice for identifying epitopes with a reasonable trade-off between sensitivity and specificity.^45^ For this study, epitope prediction was performed using ABCpred's 16-mer window length and a threshold of 0.51, leveraging its RNN architecture to refine predictions and applying an overlapping filter for enhanced accuracy.

Additionally, the Bepipred Linear Epitope Prediction 2.0 tool, accessed via the IEDB Analysis platform, was utilized to predict epitopic residues, with scores exceeding the default threshold of 0.5 considered indicative of an epitope. This threshold offers sensitivity and specificity values of 0.58564 and 0.57158, respectively, outperforming other sequence-based epitope prediction methods.^46^ Residues with scores exceeding the threshold were identified as part of a potential epitope and were labeled with an “E”. Only those residues marked “E” were selected for further analysis, allowing a concentrated evaluation of the most promising candidates for vaccine development.

### 2.5 Identification of helper T-cell epitopes

Helper T cells, or CD4+ T cells, play a critical role in vaccine development by orchestrating immune responses. They activate B cells and cytotoxic T cells, secrete key cytokines, and enhance dendritic cell function, contributing to robust and durable immunity. To predict Helper T lymphocyte (HTL) epitopes, multiple computational tools were used, including the IEDB MHC II server, RANKPEP server, and Propred-2 server. Notably, the NetMHCIIpan 4.1 EL method within the IEDB MHC II server was employed for epitope prediction, with species and locus set to Human/HLA-DR. The selected alleles for HLA class II epitope prediction were DRB1*01:01, DRB1*03:01, DRB1*04:01, DRB1*07:01, DRB1*08:01, DRB1*11:01, DRB1*13:01, and DRB1*15:01, which collectively encompass over 95% of the global human population's HLA variability.^47^ The RANKPEP server was employed to predict peptide binders to MHC II molecules using Position Specific Scoring Matrices (PSSMs).^48^ The epitopes were predicted with a variability threshold of 1 and a binding threshold top number of 990 to ensure 49.5% sensitivity, 76.0% specificity. The Propred-2 server predicted the epitopes using its default settings and displayed the top 10 peptides for the specified alleles.^49^ Peptides scoring above 0.5 in IEDB were prioritized for further analysis, ensuring a higher likelihood of binding to the target MHC molecules. In contrast, all peptides predicted by RANKPEP and Propred-2 were included for subsequent evaluation.

### 2.6 Identification of cytotoxic T-cell epitopes

Cytotoxic T cells (CD8+ T cells) are crucial for vaccine-induced immune responses, targeting and destroying infected cells to eliminate intracellular pathogens like viruses and bacteria. They recognize infected cells via antigens presented by MHC class I molecules. Vaccines include specific epitopes to prime these cells, aiming to induce long-lasting memory cytotoxic T cells for rapid response upon re-exposure. These cells offer broad protection against various pathogen strains, especially mutating viruses, by recognizing diverse peptides. Complementing antibodies, they ensure comprehensive defense by targeting intracellular pathogens, reducing viral load, and controlling outbreaks.^50^

MHC class I epitopes were discerned utilizing the IEDB MHC I server specifically tailored for the targeted protein. This server employs an Artificial Neural Network (ANN) model to forecast conserved 10-mer sequences from HLA class I T-cell epitopes.^51^ Version 2.2 of the ANN, selected for its reliance on the median inhibitory concentration (IC_50_), was utilized for prediction purposes. In the pursuit of binding analysis, human-derived MHC molecules were designated as the source species, and alleles were meticulously chosen. Peptides were subsequently sorted based on their predicted IC_50_ values. The chosen alleles for HLA class I epitope prediction encompassed those most prevalent in the global population, each exhibiting a frequency of ≥6%.^52^, ^53^ Noteworthy alleles included HLA-A*02:01, HLA-A*02:02, HLA-A*02:03, HLA-A*02:06, HLA-A*68:02, HLA- A*23:01, HLA-A*24:02, HLA-A*30:02, HLA-A*29:02, HLA-A*01:01, HLA-B*35:08, HLA- B*07:02, HLA-B*51:01, HLA-B*54:01, HLA-B*35:01, HLA-B*35:08, HLA-B*51:01, HLA- B*53:01, HLA-A*33:01, HLA-A*68:01, HLA-A*03:01, HLA-A*11:01, and HLA-A*33:01.

An inverse relationship exists between binding affinity and IC_50_, where a lower IC_50_ value corresponds to higher binding affinity of an epitope to MHC-I with HLA molecules. Specifically, IC_50_ values of ≤10 nM, ≤100 nM, and ≤1000 nM reflect strong, moderate, and weak binding affinities, respectively.^54^ To ensure the selection of immunodominant peptides with the highest potential for T-cell activation, only those with IC_50_ values ≤100 nM were included in the analysis, while peptides exceeding this threshold were excluded.^55^

### 2.7 Identification and evaluation of epitopes for B cells, helper T cells, and cytotoxic T cells

The selection of B-cell, Helper T-cell, and Cytotoxic T-cell epitopes involves a careful screening of their antigenic, toxic, and allergenic properties, along with physicochemical traits like instability index and GRAVY score. Epitopes must be highly antigenic to trigger strong immune responses, interact with MHC molecules, and be non-toxic to ensure vaccine safety. Avoiding allergenicity is vital to prevent allergic reactions. Stability is crucial; unstable epitopes degrade quickly, undermining immune response. Generally, hydrophilic epitopes are favored for their solubility, stability, and improved presentation by antigen-presenting cells, enhancing immune response.^56^

Antigenicity, toxicity, and allergenicity predictions were performed using VaxiJen v2.0, ToxinPred, and AllerTOP v2.0, respectively. VaxiJen v2.0 employs a novel alignment-free antigen prediction algorithm based on auto cross-covariance (ACC) transformation, converting protein sequences into uniform vectors. This method achieves prediction accuracies ranging from 70% to 89%. The target organism for this study was selected as bacteria, with an antigenicity threshold of 0.5 used for epitope assessment. This threshold was selected to achieve a balance between accuracy, sensitivity, and specificity, ensuring reliable identification of antigenic regions.^57^, ^58^ Furthermore, the Expasy ProtParam server was utilized to evaluate the physicochemical properties of the identified epitopes.

Epitopes meeting stringent criteria for antigenicity, lack of toxicity and allergenicity, stability, and hydrophilicity were exclusively selected for vaccine construct incorporation. This rigorous selection process ensures inclusion of epitopes with optimal immunogenic potential while minimizing potential adverse effects, thereby enhancing vaccine candidate safety and efficacy. Furthermore, the final vaccine constructs also underwent thorough evaluation to ensure adherence to these criteria.

### 2.8 Population coverage analysis

The imperative for vaccine design extends to achieving broad coverage across diverse global populations, recognizing the variation in HLA diversity among different ethnicities.^59^ In this study, the Immune Epitope Database (IEDB) tool (https://tools.iedb.org/population/) was employed to evaluate the representation of HLA class I and II alleles across diverse populations. The dataset encompasses frequencies for 115 countries, 21 ethnicities, and 16 geographical regions. Recognizing the variability in epitope binding preferences among HLA alleles, careful selection was made to identify epitopes with the highest potential for global and region-specific population coverage. The analysis utilized the “world” setting for geographic coverage and applied the “Class I separate and Class II separate” calculation option to ensure a thorough evaluation. The output included key metrics such as population coverage, HLA combinations recognized, average epitope hits, and the minimum epitope combinations required to achieve 90% population coverage (pc90).^60^

### 2.9 Construction of recombinant multi-epitope vaccine

The final vaccine sequence was assembled by integrating conserved epitopes identified through multiple immunoinformatics tools, along with suitable adjuvants and linkers. Three peptide adjuvants were selected for further analysis based on their documented capacity to enhance antigen presentation and immune response: the 50S ribosomal protein (accession no. P9WHE3), β-defensin (accession no. O15263), and HBHA (accession no. P9WIP9).^61^, ^62^, ^63^

The adjuvant 50S ribosomal protein engages TLR4, triggering the maturation of dendritic cells (DCs) and promoting the production of pro-inflammatory cytokines, including IL-1β, TNF-α, and IL-6. This process is partially regulated through both TRIF and MyD88 signaling pathways. The activation of DCs further stimulates naive T cells, effectively polarizing CD4+ and CD8+ T cells. This polarization leads to the secretion of IFN-γ and the initiation of robust T cell-mediated cytotoxic responses.^64^ β-defensins are small peptides that play a crucial role in host defense by forming mucosal barriers against pathogens.^65^ They attract immune cells by interacting with chemokine receptors like CCR6, thus enhancing the immune response to bacterial infections.^66^ β-defensins also activate TLRs on immune cells, triggering the release of pro-inflammatory cytokines, which initiate immune responses.^67^ Additionally, β-defensins promote adaptive immunity by facilitating the activation and maturation of dendritic cells, enhancing antigen presentation, and stimulating T-helper cell responses.^65^ Given its dual impact on innate and adaptive immunity, β-defensins hold potential as an adjuvant in vaccines and therapeutic strategies against bacterial infections. HBHA, a surface-expressed adhesin, has been identified as a novel TLR4 agonist and a promising vaccine adjuvant.^68^ By binding to its specific TLRs, HBHA facilitates the polarization of naive CD4+ T cells into Th1 cells, thereby inducing a strong CTL response. Furthermore, TLR activation by HBHA enhances both adaptive and innate immunity by promoting the secretion of inflammatory cytokines such as IL-1, IL-6, and IL-12, facilitating co-stimulatory signaling in antigen-presenting cells (APCs), and upregulating MHC molecules. These immunostimulatory properties position HBHA as a potent adjuvant, suitable for inclusion in multi-epitope vaccine formulations to enhance their efficacy.^69^, ^70^

The adjuvants were linked to the N-terminal using the EAAAK linker, a stable and rigid α-helical peptide linker facilitating fusion protein formation.^46^ Rigid linkers such as EAAAK are highly effective in minimizing domain movement and reducing undesirable interactions within fusion proteins.^71^ By maintaining a consistent spatial separation between epitopes, these linkers ensure functional domain independence and preserve the distinct characteristics of each epitope. This structural stability is particularly advantageous for bifunctional fusion proteins, as it facilitates efficient domain separation and enhances their functional performance.^72^

To link immunodominant CTL and HTL epitopes, AAY and GPGPG linkers were employed, respectively, due to their optimal length, flexibility, and capacity to enhance immune processing and presentation.^73^ The AAY linker serves as a specific cleavage target for proteasomes, enabling precise intracellular processing within mammalian cells. This targeted cleavage efficiently separates epitopes linked by the AAY motif, thereby mitigating junctional immunogenicity. Furthermore, the incorporation of the AAY linker in multi-epitope vaccines often enhances their overall immunogenicity, thereby increasing the potential to elicit robust and effective immune responses.^74^ The GPGPG linker serves two critical functions in the design of epitope-based vaccines. First, it prevents the formation of junctional epitopes, thereby ensuring the specificity of the immune response.^75^ Second, it facilitates the processing and presentation of target epitopes by HLA class II molecules to the immune system. Additionally, studies have demonstrated that the GPGPG linker can effectively modulate infections by eliciting HTL responses, whether through polypeptide-based or DNA vaccination approaches.^76^

The KK linker was employed to connect B-cell epitopes, providing enhanced stability and flexibility in chimeric proteins or multi-epitope constructs.^77^ By introducing a flexible spacer, this linker facilitates proper folding and presentation of epitopes while minimizing potential interference within the vaccine construct.^78^ Notably, the KK linker serves as a target sequence for cathepsin B, a lysosomal protease critical for antigen processing in the context of MHC-II antigen presentation.^79^ Peptides linked by KK linkers ensure distinct presentation of each peptide to antibodies, thereby reducing the likelihood of eliciting antibodies against the artificial amino acid sequence created by the fusion of two peptides.^80^ Furthermore, KK linkers contribute to improved immunogenicity, enhancing the overall efficacy of the vaccine design.^81^

Addition of the TLR4 agonist RS09 (APPHALS) to the C- terminal of the Multi epitope vaccine (MEV) enhances antigenicity and immunogenicity by stimulating innate immune responses via TLR4 activation, promoting APC maturation, antigen processing, and presentation, ultimately inducing robust adaptive immune responses and supporting long-lasting immune memory.^82^ Additionally, RS09 facilitates the co-stimulation of CTL epitopes, amplifying the immune response. The use of synthetic adjuvants like RS09 offers a safer and more advanced alternative to traditional vaccination approaches.^83^ To improve protein purification and identification, a hexahistidine tag (HHHHHH) was added to the protein's C-terminal. This His-tag facilitates efficient purification through affinity chromatography, using chelated metal ions as ligands. The 6 × His-tag offers several advantages, including its compact size, lack of charge, and minimal toxicity and immunogenicity.^84^

### 2.10 Prediction of secondary and tertiary structures for the recombinant vaccine

The secondary structure of the MEV was predicted using the PSIPRED Workbench server, a highly accurate tool for protein annotation and biological analysis. For this study, the entire sequence of the designed vaccine was submitted to PSIPRED 4.0, which employs advanced algorithms to deliver detailed insights into the secondary structure, enhancing our understanding of the MEV's folding and structural characteristics.^85^

Homology modeling of the multi-epitope vaccine peptide was performed using the I-TASSER server (https://zhanglab.ccmb.med.umich.edu/I-TASSER/). I-TASSER employs a sophisticated sequence-to-structure-to-function approach to predict protein structure and function. The platform identifies structural analogs from the Protein Data Bank and constructs 3D atomic models via threading alignments and iterative structural assembly simulations based on the amino acid sequence. Initially, LOMETS, a meta-server integrating 11 threading programs, generates multiple templates aligned with the query sequence. Templates with the highest z-scores from each program are selected. I-TASSER refines these templates to produce the top five models, each with a Confidence score (C-score) that reflects model reliability, with higher C-scores indicating greater confidence. Additionally, I-TASSER calculates the TM-score and Root Mean Square Deviation (RMSD) for each model, where the TM-score evaluates structural similarity (values above 0.5 signify accurate topology). The final model chosen by I-TASSER is the one with the optimal balance between the C-score and TM-score, ensuring both accuracy and reliability in the predicted structure.^86^

### 2.11 Refinement and validation of tertiary protein structure

The tertiary structure produced by I-TASSER was further optimized with GalaxyRefine2, accessible via the GalaxyWEB server (https://galaxy.seoklab.org/index.html). GalaxyRefine2, an advanced iteration of 'GalaxyRefine', leverages both local and global refinement techniques, along with local error assessments and homology-based structural information, to enhance the precision of the protein model obtained from I-TASSER's top prediction.^87^ GalaxyRefine2 produces 10 optimized models, providing comprehensive metrics such as RMSD, MolProbity scores, Clash scores, incidence of poor rotamers, Ramachandran favored regions, and 'GALAXY energy' relative to the initial model. This facilitates improved structural accuracy and reliability. The highest-quality refined model was selected for subsequent computational analyses, including docking studies and *insilico* cloning, to evaluate its potential efficacy and stability as a vaccine candidate. The refined tertiary structure, obtained using GalaxyRefine2, was validated through multiple methods.

The ProSA (Protein Structure Analysis) tool is widely regarded as a reliable method for the validation and refinement of both experimental and predicted protein structures. It is commonly used for evaluating the stability and accuracy of models by providing key insights into the energetic properties of the structure. ProSA’s Z-score and local quality profile help determine whether the protein model is physically feasible and likely to exhibit correct folding.^88^ However, it is important to acknowledge that ProSA primarily evaluates energetic aspects, and as such, it cannot offer a comprehensive assessment of the protein structure on its own. To ensure thorough validation, ProSA should be complemented by additional tools such as Ramachandran plots, Verify3D, or RMSD analysis. ProSA-web (https://prosa.services.came.sbg.ac.at/prosa.php) specifically assessed model quality of the vaccine candidates by computing the Z-score, which reflects the alignment of the model’s fold with that of experimentally determined structures. A higher Z-score suggests better sequence identity with the template and preservation of critical functional or structural residues, thereby increasing confidence in the model's accuracy.^88^

Additionally, ERRAT server (https://services.mbi.ucla.edu/ERRAT/) was used to analyze non-bonded atom interactions by comparing them to high-resolution crystallographic data. The Ramachandran plot, generated using PROCHECK, displays the allowed and disallowed dihedral angles (ψ and ϕ) for amino acids based on their van der Waals radii, providing insight into the model's structural quality.

Verify3D is a widely recognized structural validation tool that assesses the compatibility of a protein's 3D model with its corresponding amino acid sequence. It categorizes each residue based on its structural environment (e.g., alpha helix, loop, beta sheet, polar, or non-polar) and compares the results to high-quality reference structures. This method is particularly valuable for evaluating protein structures derived from computational approaches like homology modeling.^89^, ^90^ However, to ensure robust structural validation, Verify3D should be integrated into a comprehensive validation strategy, complementing tools that assess energy stability, atomic packing, and geometric integrity. When combined with other methods, Verify3D enhances the reliability of protein models. In this study, Verify3D (https://www.doe-mbi.ucla.edu/verify3d/) was utilized to confirm that the amino acid residue profiles of the vaccine constructs’ 3D models align with typical structural characteristics.^89^

### 2.12 Molecular docking of the optimized vaccine candidate with immune receptors

Molecular docking is a computational technique used to assess the interaction between proteins or between a small molecule and its receptor, generating a simulated score that reflects binding affinity and interaction. The efficacy of an immune response relies on the precise engagement of antigenic molecules with specialized immune receptors. In this study, protein–protein docking was performed to determine the binding affinity between the vaccine construct and key antigenic recognition receptors, specifically MHC-I, MHC-II, TLR2, and TLR4.

Docking of the MEV-immune receptor was conducted using the HDOCK server, which generated the top 10 models ranked by binding energy scores. The model with the lowest energy was chosen for subsequent analysis. HDOCK utilizes a hybrid approach, integrating both template-based and template-free docking techniques to predict receptor-ligand interactions.^91^ Protein-protein docking was also carried out using the ClusPro server, generating 10 models for each docked complex. ClusPro utilizes the Piper rigid body program, rotating the vaccine construct and receptor protein across the x, y, and z axes with 70,000 rotations and 1.0 Å spacing.^92^ Optimal models were identified based on weighted scores indicating the lowest energy and visualized using PyMOL. Hydrogen bonds, salt bridges, and other non-covalent interactions were evaluated using the PDBsum server. Subsequently, the docked MEV-immune receptor complexes underwent molecular dynamics simulations for 100 ns.

### 2.13 Molecular dynamics simulation

The vaccine constructs and complexes exhibiting robust binding affinity to core targets were subjected to molecular dynamics (MD) simulations to evaluate their binding stability and flexibility. These simulations were essential for assessing the stability and conformational dynamics of the proposed models. Additionally, the interactions between the MEV construct and immune receptors were analyzed at different time points.

MD simulations were performed using the GROMACS 2023 package,^93^ spanning 100 ns and encompassing the MEV construct, immune receptors, and their complexes. The GROMOS 54a7 force field was employed to generate protein topologies, and all systems were solvated in a cubic water box using the SPC/E water model, ensuring a minimum distance of 1.0 nm between solute molecules and the box boundary. To neutralize the net charge, counter ions (sodium or chloride) were introduced.^94^ The Verlet cutoff scheme was applied for neighbor list generation, while long-range electrostatic interactions were calculated using the particle mesh Ewald (PME) method.^95^, ^96^ Covalent bond lengths were constrained with the LINCS algorithm,^97^ and the integration step was set to 2 fs. The system was energy-minimized over 50,000 steps using the steepest descent method and equilibrated in two phases: an initial 2 ns NVT simulation to stabilize temperature, followed by a 5 ns NPT simulation to equilibrate pressure, maintaining a fixed number of particles, pressure, and temperature. The production phase consisted of a 100 ns NPT simulation at 300 K and 1 bar.^98^ Temperature and pressure were regulated using the modified Berendsen thermostat and the Parrinello-Rahman barostat, respectively.^99^, ^100^ Simulation data were analyzed for structural stability and dynamics, including protein root mean square deviation (RMSD), root mean square fluctuations (RMSF), and the radius of gyration (Rg), using the Xmgrace program.^101^

### 2.14 Prediction of discontinuous B-cell epitopes

Most B-cell epitopes are conformational, formed by 1–5 linear amino acid segments that are non-contiguous in sequence but come together in close proximity in three-dimensional space to interact with antibodies. Approximately 90% of B-cell epitopes are discontinuous, comprising segments that are distantly located in the primary sequence but are brought into proximity through protein folding.^102^ The engineered MEVs undergo folding to form conformational B-cell epitopes, which are vital for triggering an adaptive immune response. Accurate prediction of these discontinuous B-cell epitopes is critical for optimizing the spatial structure of MEVs. To facilitate this, we employed the ElliPro server (https://tools.iedb.org/ellipro/), for predicting nonlinear B-cell epitopes. ElliPro utilizes three algorithms: approximating the protein's shape as an ellipsoid, calculating the residue protrusion index (PI), and clustering adjacent residues based on PI values. Epitope scoring is based on the PI value averaged over its residues. For example, an ellipsoid with a PI value of 0.9 encompasses 90% of the protein residues, with the remaining 10% outside. Residues are scored according to their distance from the ellipsoid's center of mass.^103^ Conformational B-cell epitopes with a prediction score greater than 0.69 are considered acceptable, indicating a high likelihood of proper folding and effective antibody interaction.

### 2.15 Codon optimization and computational cloning of the recombinant vaccine

After identifying the most promising vaccine candidate through immunoinformatics analysis, the JAVA Codon Adaptation Tool (JCat) (https://www.jcat.de/Start.jsp) was utilized for reverse translation and codon optimization to enhance the expression of the vaccine candidate in *E. coli* (strain K12). Codon optimization is crucial for improving gene expression in the chosen expression vector and host cell. In the JCat tool, settings were adjusted to avoid rho-independent transcription terminators, prokaryotic ribosome binding sites, and restriction enzyme cleavage sites. The output included the codon adaptation index (CAI) and GC-content of the optimized sequence.^104^

CAI is a quantitative metric used to evaluate how well the codon usage of a gene aligns with the preferred codons of a reference set, typically derived from highly expressed genes. The CAI score ranges from 0 to 1, with higher scores indicating a greater degree of adaptation to the optimal codon usage of the reference set. Genes with higher CAI scores are associated with improved translation efficiency and enhanced protein expression levels. A score nearing 1.0 is considered optimal, while values above 0.8 are deemed excellent for achieving efficient translation and robust protein production.^105^ The optimal GC content for recombinant genes typically ranges from 30% to 70%. Deviations outside this range may adversely affect transcription and translation efficiency and can lead to structural or biochemical challenges, compromising gene expression and function.^106^ This balance of codon adaptation and GC content is critical for maximizing the efficiency and success of recombinant protein expression systems.^107^

The codon-optimized nucleotide sequence for the final recombinant vaccine construct was designed and seamlessly integrated into the *E. coli* pET28a(+) vector using SnapGene software. The pET system is widely regarded as a robust platform for the cloning and expression of recombinant proteins, particularly multi-epitope vaccines, in *E. coli*. Its versatile design, featuring multiple cloning sites and support for sticky-end cloning, facilitates the efficient incorporation of complex vaccine constructs. Additionally, the system's capacity for high protein yields makes it a preferred choice for multi-epitope vaccine development.^108^

To optimize the cloning process, XhoI and NdeI restriction sites were strategically incorporated at the C-terminal and N-terminal ends of the construct, respectively. These sites create sticky ends with complementary sequences, enabling efficient annealing and ligation. Importantly, this approach allowed for directional cloning, ensuring the correct orientation of the insert while minimizing the risk of vector self-ligation. By employing this strategy, the overall cloning efficiency was enhanced, ensuring accurate expression of the vaccine construct.^109^

### 2.16 Immune simulation

To predict the immunogenicity and immune response characteristics of the chimeric peptide, we performed *in silico* immune simulations using the C-ImmSim server. C-ImmSim utilizes an agent-based model that integrates a PSSM for immune epitope prediction and machine learning algorithms for predicting immune interactions. The simulation framework includes three compartments corresponding to key anatomical regions in mammals: the bone marrow, where hematopoietic stem cells produce lymphoid and myeloid cells; the thymus, where naive T cells are selected to avoid autoimmunity; and a tertiary lymphoid organ, such as a lymph node.^110^ The simulation was performed using default parameters with an injection time step of 1, representing 8 h of daily life per step. The simulation volume was set to 10, with a total of 100 steps and 1000 antigens injected. Vaccine was selected as the injection type without LPS (Lipopolysaccharide), reflecting the composition of the tested vaccine, and the adjuvant concentration was set to 100.

## 3 Results and discussion

### 3.1 Subtractive genomic analysis

The current study employs an advanced subtractive genomics approach to identify potential vaccine targets. An *in silico* analysis was performed, revealing that the complete proteomes of *Homo sapiens* and *Streptococcus pyogenes* consist of 136,194 and 1602 proteins, respectively. Using the CD-HIT tool, it was determined that 8 out of the 1602 proteins in *S. pyogenes* were paralogous, leaving 1594 non-paralogous proteins for further investigation. The non-paralogous proteins were analyzed using BLASTp against the human proteome to identify and exclude those with homology to human proteins, thereby minimizing the risk of cross-reactivity and potential toxicity in humans. The BLASTp results showed that 1260 proteins had similarities with human proteins, thus were excluded from further analysis. Consequently, 334 non-homologous proteins were selected for downstream analysis.

The subsequent step involved performing a TBLASTN search against the DEG database, which comprises a comprehensive collection of essential genes from diverse pathogenic and non-pathogenic organisms, including both prokaryotes and eukaryotes. This search identified 71 proteins as essential for the viability of *S. pyogenes*, making them potential vaccine targets. These 71 essential proteins were then mapped to UniProt IDs, resulting in a total of 314 hits. Among these, 110 hits were reviewed entries, and 204 were unreviewed. For further analysis, the 110 reviewed hits were selected due to their manual curation by experts, ensuring high-quality data. The UniProt ID mapping results are detailed in Table S1 of the Supplementary Information, SI section and the detailed step-by-step filtering process of the proteins in this study is shown in Table 1.

**Table 1:** Proteins identified in this study: Subtractive filtering of *S. pyogenes* proteins.

Sl no.	Steps Involved in Current Study	*H. Sapiens* Proteins	*S. Pyogenes* Proteins
1	Complete proteome of *H. sapiens & S. pyogenes*	136194	1602
2	Number of proteins left after CD-HIT	33625	1594
3	BLASTP of proteins against human host proteome (E value 10^−5^)		334
4	TBLASTN of non-homologous proteins against DEG (E value 10^−100^)		71
5	No. of hits after ID Mapping		314

### 3.2 Prediction of subcellular localization

Using the subtractive genomic approach, the BUSCA tool was utilized to determine the subcellular localization of non-homologous essential proteins. From the initially identified pool of 110 potential vaccine targets, a curation process was conducted to eliminate redundant proteins. Among the remaining 30 proteins, 86.67% were predicted to localize within the cytoplasm, while only 13.33% were anticipated to reside in the plasma membrane, as indicated in SI Table S2. The plasma membrane proteins were prioritized for further scrutiny due to their distinct advantages in vaccine development. These proteins, situated on the cell surface, exhibit facile recognition by the immune system, thereby eliciting targeted immune responses. Their uniqueness in pathogens reduces the likelihood of cross-reactivity with host proteins, thus minimizing autoimmune risks. Vaccines targeting such proteins prompt the production of neutralizing antibodies, impeding pathogen invasion and infection. Furthermore, they induce enduring immune memory and can be incorporated into multivalent vaccine formulations, augmenting efficacy and diminishing the emergence of vaccine-evading mutants.^111^ Consequently, the plasma membrane proteins atpF, dnaA, hrcA, and ezrA emerged as promising vaccine candidates.

The roles of atpF, dnaA, hrcA, and ezrA in the biology and pathogenesis of *Streptococcus pyogenes* are pivotal, as they regulate essential cellular processes. atpF functions as a constituent of ATP synthase, facilitating ATP biosynthesis vital for metabolic activities and cell division.^112^ dnaA orchestrates DNA replication by binding to specific genetic sequences, thus enabling the duplication of chromosomes crucial for cellular proliferation.^113^ hrcA operates as a transcriptional modulator, regulating the expression of heat shock proteins (HSPs) to fortify bacterial survival under thermal stress conditions by safeguarding and repairing cellular constituents.^114^ ezrA actively participates in cell division and the synthesis of the cell wall, thereby ensuring cellular morphology and structural integrity through its interactions with other division-related proteins.^115^ Collectively, these proteins coordinate fundamental cellular activities encompassing energy generation, DNA replication, stress response, and cellular division, thereby influencing the persistence and pathogenicity of *Streptococcus pyogenes.*

### 3.3 Antigenic, allergenic and toxicity prediction of the proteins

To prioritize candidates for the final recombinant vaccine, a comprehensive evaluation of four surface proteins was conducted, focusing on their antigenicity, allergenicity, and toxicity. Proteins demonstrating high antigenic potential without allergic or toxic properties were prioritized to ensure both safety and efficacy as vaccine antigens.

Among the proteins analyzed, atpF emerged as the most promising target. ANTIGENpro analysis revealed that atpF exhibited the highest antigenicity score (0.927767) compared to dnaA (0.643495), hrcA (0.261506), and ezrA (0.770638), underscoring its superior potential as a robust vaccine candidate. Furthermore, subsequent assessments using AllerTOP v.2 and ToxinPred 2 confirmed that all four proteins, including atpF, were non-allergenic and non-toxic, thereby validating their suitability for vaccine development. The prioritization of atpF was driven by its exceptional antigenicity score, which significantly surpassed the other candidates, making it a prime target for subsequent analyses. Detailed comparative data for antigenicity, toxicity, and allergenicity of all proteins are presented in SI Table S3.

### 3.4 Prediction of linear B-cell epitopes and T-cell epitopes

Identifying T-cell and B-cell epitopes is paramount in devising effective vaccine strategies. T-cells, categorized into CD4+ (HTLs) or CD8+ (CTLs) based on their surface receptors, engage with epitopes presented by MHC-I and MHC-II molecules. CTLs recognize MHC-I binding epitopes, while HTLs interact with MHC-II molecules. Both CTLs and HTLs play pivotal roles in orchestrating long-lasting adaptive immunity against diverse microbial threats. CTL epitopes contribute to cellular immunity, facilitating the elimination of infected cells and circulating antigens.^116^ In contrast, HTL epitopes are crucial for inducing both humoral and cell-mediated immune responses.^117^ Notably, HTL epitopes are crucial for generating memory helper T cells, which are essential for activating cytotoxic T cells and stimulating B lymphocytes to produce antibodies.^118^ Consequently, the inclusion of HTL and CTL epitopes is preferred in designing efficacious vaccine candidates capable of eliciting robust antibody responses and cytotoxic activity.

#### 3.4.1 B-cell epitope prediction

Linear B-cell epitopes of the atpF protein, exhibiting varying residue lengths, were identified using the ABCpred and IEDB prediction servers. To ensure the inclusion of epitopes with the highest immunogenic potential, the predicted epitopes were subjected to a rigorous screening process based on antigenicity. ABCpred identified 13 epitopes, and IEDB predicted 5, yielding a total of 18 candidate epitopes (SI Tables S4 and S5). Epitopes demonstrating high antigenicity were further shortlisted, resulting in 15 epitopes that underwent detailed analysis. These shortlisted epitopes were evaluated using the instability index and GRAVY scores to assess their biochemical stability and hydrophilicity, parameters critical for their effective recognition and elicitation of an immune response. From this comprehensive evaluation, six epitopes (DKLVAEATDEAKRLKE, AEKIMGANLDKTAQSQ, ELVG, QLQGDKLVA, KRLKEKALT, and KRKEKLTDE) were selected, as they consistently exhibited superior antigenicity, stability, and hydrophilicity.

The inclusion of these six epitopes was intended to achieve a balanced and robust immunogenic response by leveraging their diverse biochemical and immunological properties. Reducing the number of epitopes was carefully considered; however, it was determined that the selected combination of six epitopes would maximize immune coverage while minimizing the risk of unbalanced or suboptimal immunogenicity. This approach ensures that the vaccine construct provides comprehensive and effective immune protection. Table 2 provides a detailed list of the identified B-cell epitopes.

**Table 2:** Identification of B-cell epitopes and prediction of their stability and hydropathicity. Epitopes that were selected and incorporated into the final vaccine construct are highlighted in bold.

Peptide	Length	GRAVY^a^	Instability Index^b^	
			Score	Classification
**DKLVAEATDEAKRLKE**	**16**	**−1.075**	**13.83**	**stable**
TRSQQISRDIDQAEQS	16	−1.613	112.45	unstable
SRDIDQAEQSRLSAQQ	16	−1.500	84.99	unstable
SDAISAVKTEMSDLTV	16	0.250	47.07	unstable
DKTAQSQLIDSYLDDL	16	−0.675	55.61	unstable
**AEKIMGANLDKTAQSQ**	**16**	**−0.725**	**32.96**	**stable**
AKSQANLDASRSQASK	16	−1.156	45.49	unstable
DIEQSKSDAISAVKTE	16	−0.725	86.58	unstable
SRLSAQQLEAKSQANL	16	−0.719	45.49	unstable
RSQQISRDIDQAEQ	14	−1.736	95.70	unstable
**ELVG**	**4**	**−0.133**	**−25.96**	**stable**
**QLQGDKLVA**	**9**	**−0.133**	**26.24**	**stable**
LDKTAQSQLIDS	12	−0.525	70.81	unstable
**KRLKEKALT**	**9**	**−1.222**	**35.68**	**stable**
**KRKEKLTDE**	**9**	**−2.622**	**35.68**	**stable**

a- A higher positive GRAVY value signifies increased hydrophobicity, whereas negative values indicate hydrophilicity.

b- Proteins with an instability index below 40 are classified as stable, while those with a value exceeding 40 are considered potentially unstable.

#### 3.4.2 T-cell epitope prediction

CTL epitopes were predicted utilizing the IEDB MHC I server. The server identified 7479 potential epitopes across the selected alleles. To refine the selection, only epitopes with IC_50_ values of 100 nM or lower were considered for subsequent analysis, as detailed in SI Table S6. These MHC-I epitopes were subsequently evaluated for their antigenicity. Among the 54 epitopes with IC_50_ values ≤100 nM, only 13 were found to be antigenic. These 13 epitopes underwent additional evaluation for their instability index and GRAVY scores to determine their stability and hydrophilicity. From this group, only 2 epitopes exhibited both stability and hydrophilic properties. These two epitopes were integrated into the development of a multi-epitope vaccine. Table 3 details the predicted CTL epitopes that were incorporated into the final vaccine formulation.

**Table 3:** Predicted MHC-I epitopes, along with their sequence length, and analysis of their instability and hydropathicity. Epitopes that were selected and incorporated into the final vaccine construct are highlighted in bold.

**Peptide**	**Length**	**GRAVY^a^**	**Instability Index^b^**	
			**Score**	**Classification**
ELVGNFILV	9	1.767	−5.28	stable
**KIMGANLDK**	**9**	**−0.356**	**−19.41**	**stable**
DASRSQASK	9	−1.578	100.51	unstable
ASRSQASKI	9	−0.689	91.08	unstable
MSITFGELV	9	1.311	63.04	unstable
SVIVLLLLIK	10	2.790	−7.98	stable
VIVLLLLIK	9	3.189	−9.98	stable
LLIKKFAWGA	10	0.940	−18.35	stable
KSQANLDASR	10	−1.310	47.52	unstable
KTEMSDLTVL	10	0.060	72.20	unstable
TAQSQLIDSY	10	−0.400	91.46	unstable
QTRSQQISR	9	−1.922	121.91	unstable
**LEAKSQANL**	**9**	**−0.444**	**30.29**	**stable**

a- A higher positive GRAVY value signifies increased hydrophobicity, whereas negative values indicate hydrophilicity.

b- Proteins with an instability index below 40 are classified as stable, while those with a value exceeding 40 are considered potentially unstable.

High-affinity MHC-II epitopes specific to human HLA-DR alleles were identified using the IEDB MHC-II, RANKPEP, and ProPred 2 web servers, and subsequently classified as HTL epitopes. The IEDB server identified a total of 976 epitopes for the specified alleles, ranked based on their confidence scores. Epitopes with scores exceeding 0.5 underwent further scrutiny for their antigenic attributes, as detailed in SI Table S7. ProPred 2 and RANKPEP predicted 61 and 15 epitopes, respectively (SI Table S8 and S9). These predictions were also subjected to antigenicity analysis, and epitopes demonstrating significant antigenic potential were subsequently assessed for their instability index and GRAVY scores to ascertain stability and hydrophilicity. Among the selected epitopes, only four exhibited both stability and hydrophilic characteristics. The four epitopes were subsequently incorporated into the formulation of a multi-epitope vaccine. Table 4 details the HTL epitopes selected for inclusion in the final vaccine design.

**Table 4:** Predicted MHC class-II epitopes, along with their sequence length, and analysis of their instability and hydropathicity. Epitopes that were selected and incorporated into the final vaccine construct are highlighted in bold.

**Peptide**	**Length**	**GRAVY^a^**	**Instability Index^b^**	
			**Score**	**Classification**
LVGNFILVT	9	2.078	−14.71	stable
VIVLLLLIK	9	3.189	−9.98	stable
VTGSVIVLL	9	2.533	−19.41	stable
LLIKKFAWG	9	0.844	−12.07	stable
**LQGDKLVAE**	**9**	**−0.133**	**26.24**	**stable**
**AKSQANLDA**	**9**	**−0.667**	**30.29**	**stable**
LLLIKKFAW	9	1.311	−0.54	stable
**SAQQLEAKS**	**9**	**−0.956**	**30.29**	**stable**
ANLDKTAQS	9	−0.944	48.28	unstable
AVKTEMSDL	9	−0.078	84.15	unstable
LQTRSQQIS	9	−1.000	136.73	unstable
**AKRLKEKAL**	**9**	**−0.944**	**35.68**	**stable**
LDASRSQAS	9	−0.722	100.51	unstable

a- A higher positive GRAVY value signifies increased hydrophobicity, whereas negative values indicate hydrophilicity.

b- Proteins with an instability index below 40 are classified as stable, while those with a value exceeding 40 are considered potentially unstable.

### 3.5 Estimated population coverage

The distribution and expression of HLA alleles vary considerably among ethnic groups and geographic regions, directly influencing the effectiveness and broad applicability of epitope-based vaccines. To evaluate the population coverage of selected CTL and HTL epitopes in relation to their corresponding HLA alleles, the IEDB population coverage tool was utilized.

**Fig. 1:**
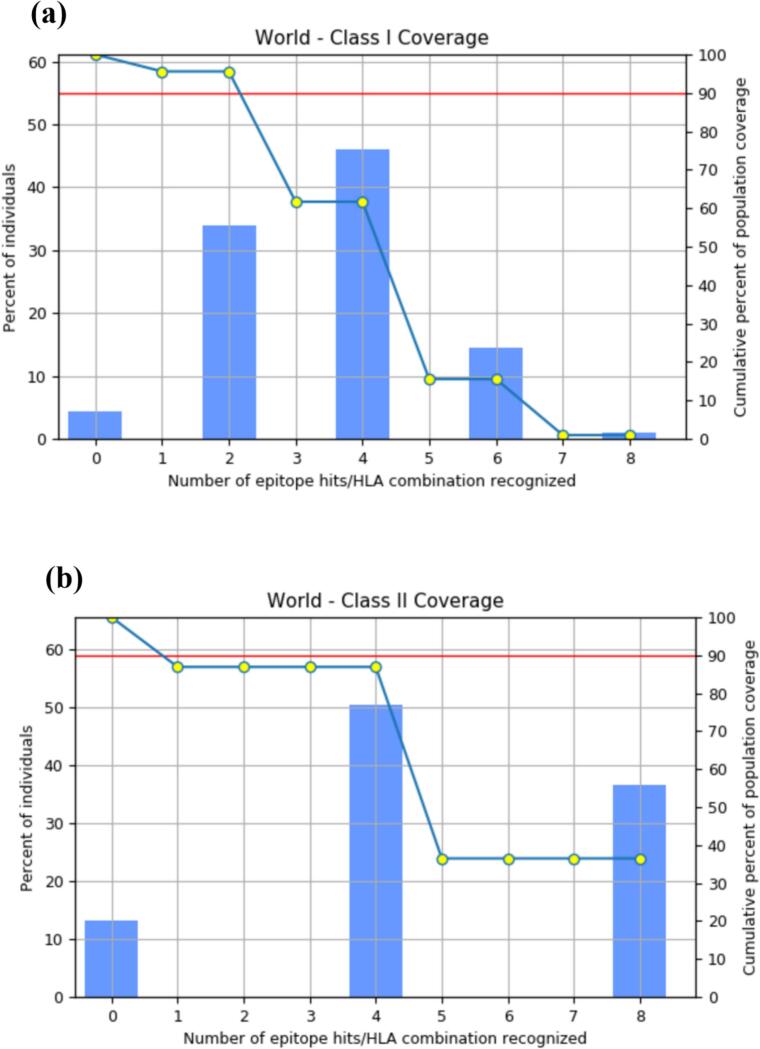
Graphical representation of the relationship between the proportion of individuals and the number of epitope-HLA allele interactions predicted for recognition: (a) MHC-I; (b) MHC-II. The histograms depict the fraction of the population capable of recognizing specific numbers of epitope-HLA combinations, with the x-axis representing the number of unique epitope-HLA allele combinations predicted per individual. The left y-axis denotes the percentage of individuals recognizing a given number of epitope-HLA interactions, while the right y-axis presents cumulative population coverage, reflecting the proportion of individuals predicted to recognize at least the specified number of combinations. The blue line represents the cumulative population coverage trend, providing insights into the distribution of epitope recognition across the population. The red horizontal line marks the 90% cumulative coverage threshold (pc90 value), indicating the level at which a sufficient proportion of the population is expected to mount an immune response. (For interpretation of the references to colour in this figure legend, the reader is referred to the web version of this article.)

The global population coverage for MHC-I epitopes was 95.6%, indicating that most individuals carry at least one HLA allele capable of presenting the selected epitopes. On average, 3.48 peptide-HLA combinations were recognized, with a pc90 value of 2.33, representing the minimum number of peptide-HLA combinations predicted to be recognized by 90% of the population. This high coverage underscores the potential of the selected MHC-I epitopes to effectively stimulate cytotoxic T-cell responses across diverse populations. For MHC-II epitopes, the population coverage was 86.92%, signifying that a significant proportion of individuals possess at least one HLA allele capable of presenting these epitopes. Each individual recognized an average of 4.94 peptide-HLA combinations, with a pc90 value of 3.06. Although slightly lower than MHC-I, this coverage demonstrates strong potential for eliciting helper T-cell responses in a wide demographic range. Fig. 1 presents the global population coverage achieved by the selected epitopes, highlighting their potential to elicit immune responses across diverse demographic groups.

While the global population coverage of the proposed vaccine is promising, it is essential to address the limitations posed by the extensive polymorphism of MHC molecules, particularly in the peptide-binding regions. This polymorphism leads to considerable variability in HLA allele distribution and binding specificities across different ethnic groups, potentially resulting in an ethnically skewed population coverage. In designing peptide-based vaccines, the uneven representation of HLA alleles could lead to an overestimation of the vaccine's efficacy across genetically diverse populations.^59^ To mitigate these challenges, it is critical to validate the predicted population coverage using experimental methods such as T-cell activation assays, and to test vaccine candidates on cell lines or primary cells expressing HLA alleles specific to diverse and underrepresented populations.

### 3.6 Designing of multi-epitope peptide vaccine

Three vaccine candidates, designated SM1, SM2, and SM3, were constructed by sequentially integrating an adjuvant, CTL epitopes, HTL epitopes, BCL epitopes, a TLR4 agonist (APPHALS), and a 6 × His-tag (HHHHHH). Each vaccine protein contained 2 CTL epitopes, 4 HTL epitopes, and 6 BCL epitopes. The primary difference among SM1, SM2, and SM3 was the adjuvant sequence used: beta defensin for SM1, 50S ribosomal L7/L12 protein for SM2, and HBHA protein for SM3.

**Fig. 2:**
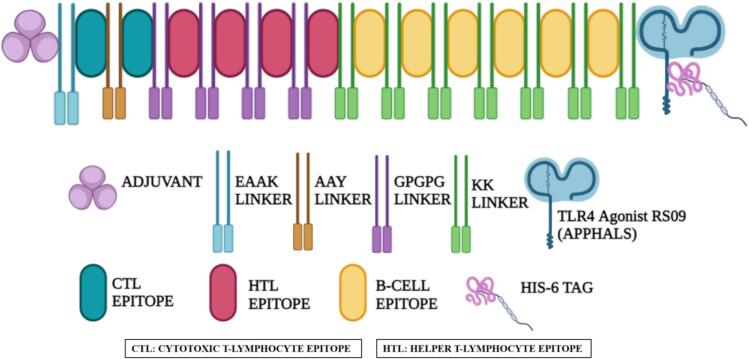
Construction of a multi-epitope vaccine. A multi-epitope vaccine construct is engineered with an adjuvant conjugated through an EAAAK linker. This is followed by the sequential incorporation of two cytotoxic T lymphocyte epitopes linked by AAY linkers. Four helper T lymphocyte epitopes are then joined using GPGPG linkers. Additionally, six B-cell epitopes are connected via KK linkers. The construct is finalized with the attachment of a TLR4 agonist and a hexa-histidine (6x-His) tag.

The construction process involved linking the adjuvant sequence to the CTL epitopes via EAAAK linkers, which provide structural stability. AAY linkers were used to conjugate CTL epitopes, markedly improving the expression and immunogenicity of the target proteins. GPGPG linkers were employed to connect CTL epitopes to HTL epitopes and to link HTL epitopes together. This linker is vital for reducing junctional immunogenicity, which arises from alterations in epitope immunogenicity, and has been shown to elicit helper T-cell responses essential for a multi-epitope vaccine. KK linkers were applied to connect HTL epitopes to BCL epitopes, BCL epitopes to one another, and BCL epitopes to the TLR4 agonist RS09 sequence. This linker minimizes junctional immunogenicity by preventing the formation of antibodies against the peptide sequences generated by linear epitope connections. Collectively, these linkers are crucial for providing structural conformation and flexibility, facilitating protein folding, separating protein domains, and enhancing the overall stability of the recombinant multi-epitope vaccine.^71^ To enhance protein purification and identification, a 6 × His-tag (HHHHHH) was attached to the C-terminus of each construct, optimizing the downstream processing of the vaccine candidates. The schematic depiction of the final multi-epitope vaccine is shown in Fig. 2.

### 3.7 Evaluation of physicochemical characteristics, antigenic profile, allergenicity, and toxicity of vaccine constructs

The antigenicity, allergenicity, and toxicity of the vaccine constructs were assessed utilizing the VaxiJen, AllerTop, and ToxinPred servers, respectively. Furthermore, the ProtParam server was employed to evaluate the physicochemical properties of the vaccines.

#### 3.7.1 Antigenicity, allergenicity, and toxicity

The antigenicity, allergenicity, and toxicity of the vaccine constructs were assessed to ensure their potential for immune activation, safety, and lack of harmful effects. The VaxiJen server predicted all three vaccine constructs (SM1, SM2, SM3) to be antigenic, with antigenicity scores of 0.8850, 0.7217, and 0.7801, respectively. These scores confirm that each construct is likely to provoke an immune response. AllerTop and ToxinPred servers evaluated the constructs as non-allergenic and non-toxic, further supporting their potential for safe application in immunization.

#### 3.7.2 Physicochemical properties

The physicochemical properties of the vaccine constructs were assessed to determine key characteristics relevant to their stability and functionality. The designed vaccine candidates, SM1, SM2, and SM3, consist of 213, 302, and 371 amino acid residues, respectively, with corresponding molecular weights of 22,656.47 Da, 31,762.72 Da, and 39,856.70 Da. The theoretical isoelectric points (pI) of these constructs varied between 9.32 and 9.88. This implies that in environments where the pH is lower than the pI, the proteins will exhibit a positive charge, whereas they will carry a negative charge in conditions where the pH exceeds their pI. This characteristic could influence their stability in different pH environments during vaccine administration.

The instability indices, which reflect the overall stability of the protein, ranged from 21.19 to 35.07 for SM1, SM2, and SM3, all of which indicate that the constructs are stable. Furthermore, the aliphatic index, which measures the relative volume of aliphatic side chains in the protein, ranged from 68.45 to 88.24, highlighting the constructs' stability across a variety of temperatures. Negative GRAVY values for the vaccine candidates (SM1: −0.878, SM2: −0.528, SM3: −0.811) suggest that the constructs are hydrophilic,^119^ promoting effective interactions with water and blood, and enabling efficient transportation through the circulatory system to target sites.

The stability of the vaccine constructs was further confirmed by their estimated half-lives in different biological systems. *In vitro*, the constructs exhibit an estimated half-life of approximately 30 h in mammalian reticulocytes, while *in vivo*, they demonstrate over 20 h of stability in yeast and more than 10 h in *Escherichia coli*. These physicochemical parameters affirm the substantial stability of the constructs in various biological environments and their compliance with standard formulation requirements, supporting their potential as vaccine candidates. SI Table S10 summarizes the key physicochemical characteristics, antigenicity, and stability parameters of the three vaccine constructs.

### 3.8 Prediction of secondary structure for the constructed vaccines

**Fig. 3:**
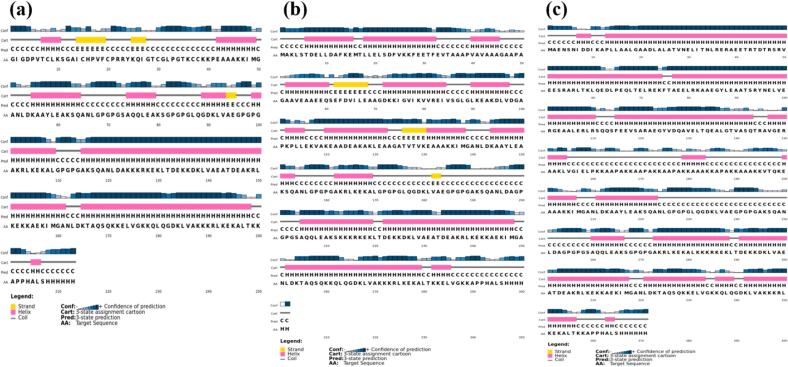
Prediction of the secondary structure for the designed *S. pyogenes* vaccines. (a) SM1, (b) SM2, and (c) SM3.

Based on the analyses conducted utilizing PSIPRED, the secondary structural composition of the vaccine constructs was determined. For SM1, 35% of the amino acid residues were observed in coil conformation, while 60% were found in the alpha helix configuration, with the remaining 5% adopting beta sheet conformation. Similarly, SM2 exhibited 36% of amino acids in coil structure, with 60% residing in the alpha helix conformation, and a minimal 4% allocation to the beta sheet structure. Conversely, SM3 showcased a comparable distribution, with 36% of amino acids in coil structure and 60% adopting the alpha helix conformation. The secondary structure predictions of the vaccine constructs are depicted in Fig. 3.

### 3.9 Prediction of tertiary structure for the vaccine construct

The tertiary structures of the vaccine constructs were determined using computational modeling with the I-TASSER web server. For each vaccine sequence, I-TASSER produced five distinct three-dimensional models. These models were ranked according to their C-scores, which gauge the confidence in the predicted models’ quality. The C-score ranges from −5 to 2, with higher scores reflecting greater confidence and more accurate structural predictions. This score is based on the significance of threading template alignments and the extent of coverage of the query sequence.

To further validate the models, multiple evaluation metrics were employed. Z-scores assessed the statistical significance of the predicted models relative to a distribution of random models. ERRAT scores evaluated the quality of non-bonded atomic interactions, while Verify-3D scores examined the compatibility between the 3D model and its amino acid sequence, focusing on residue environments. Additionally, Ramachandran plot values were used to evaluate the stereochemical quality by analyzing the dihedral angles of the amino acid residues.

The construct model that showed consistently acceptable and optimal values across all evaluated parameters, including C-score, z-score, ERRAT score, Verify-3D percentage, and Ramachandran plot values, was selected for further analysis. Detailed results of the evaluation of the vaccine constructs SM1, SM2, and SM3, conducted using the QMEAN Z-score, Ramachandran Plot, and Verify3D assessments are provided in Table 5, Table 6, Table 7.

**Table 5:** Assessment of the quality of SM1 based on QMEAN Z-Score, ERRAT score, Ramachandran Plot, and Verify3D metrics. The model selected for further analysis is highlighted in bold.

**Sl no.**	**Model name**	**Z-score**	**Ramachandran Plot**			**Verify 3D (%)**	**C-score**	**ERRAT score**
			**favoured**	**allowed**	**outlier**			
1	model 1	−4.19	58.0 %	27.0 %	4.6 %	76.53 %	−3.08	83.4146
**2**	**model 2**	**−2.44**	**78.2 %**	**17.8 %**	**1.1 %**	**61.03 %**	**−3.63**	**98.0488**
3	model 3	−4.33	76.4 %	17.8 %	1.7 %	69.48 %	−3.47	77.0732
4	model 4	−3.46	70.1 %	23.0 %	0.6 %	70.89 %	−3.85	80.4878
5	model 5	−4.83	86.2 %	10.3 %	1.1 %	58.22 %	−4.03	93.6585

**Table 6:** Assessment of the quality of SM2 based on QMEAN Z-Score, Ramachandran Plot, ERRAT score and Verify3D metrics. The model selected for further analysis is highlighted in bold.

**Sl No**	**Model name**	**Z-Score**	**Ramachandran Plot**			**Verify 3D (%)**	**C-Score**	**ERRAT score**
			**favoured**	**allowed**	**outlier**			
**1**	**Model 1**	**−3.36**	**81.2 %**	**16.5 %**	**1.1 %**	**54.64 %**	**−2.71**	**98.6395**
2	Model 2	−1.45	78.9 %	18.0 %	1.5 %	42.38 %	−3.71	71.4286
3	Model 3	−5.68	79.7 %	14.2 %	2.7 %	66.89 %	−3.04	94.8980
4	Model 4	−5.64	80.8 %	14.2 %	2.3 %	56.29 %	−2.93	95.5782
5	Model 5	−5.74	80.8 %	15.7 %	1.1 %	54.30 %	−2.97	97.9592

**Table 7:** Assessment of the quality of SM3 based on QMEAN Z-Score, ERRAT score, Ramachandran Plot, and Verify3D metrics. The model selected for further analysis is highlighted in bold.

**Sl No**	**Model name**	**Z-Score**	**Ramachandran Plot**			**Verify 3D (%)**	**C-Score**	**ERRAT score**
			**favoured**	**allowed**	**outlier**			
1	Model 1	−3.94	46.3 %	36.0 %	3.0 %	36.93 %	−2.53	73.6695
**2**	**Model 2**	**−2.14**	**71.3 %**	**19.2 %**	**4.0 %**	**63.61 %**	**−2.59**	**89.2562**
3	Model 3	−4.05	47.9 %	38.1 %	2.4 %	43.67 %	−2.88	84.8485
4	Model 4	−0.92	60.7 %	28.7 %	4.6 %	43.67 %	−3.91	87.6033
5	Model 5	−2.02	64.3 %	24.1 %	4.9 %	57.68 %	−3.99	84.8485

Table 5 presents a comprehensive assessment of five vaccine constructs, each incorporating beta defensin as an adjuvant, evaluated using metrics such as QMEAN Z-Score, Ramachandran Plot distribution, Verify3D percentage, C-Score, and ERRAT score. **Model 2** stands out as the best overall, with a QMEAN Z-Score of −2.44, 78.2% of residues in favored regions, 17.8% in allowed regions, and 1.1% outliers on the Ramachandran Plot, a Verify3D score of 61.03%, a C-Score of −3.63, and the highest ERRAT score of 98.0488. Other models show varying degrees of quality, with Model 1 having lower quality indicators and Model 5 exhibiting the best stereochemical quality but the lowest QMEAN Z-Score.

In Table 6, Model 1 demonstrates moderate overall quality with a QMEAN Z-Score of −3.36, 81.2% of residues in favored regions, a Verify3D agreement of 54.64%, a C-Score of −2.71, and an excellent ERRAT score of 98.64. Model 2 shows higher QMEAN Z-Score of −1.45 and good stereochemical quality but lower Verify3D agreement at 42.38% and a moderate ERRAT score of 71.43. Model 3, despite a low QMEAN Z-Score of −5.68, exhibits good stereochemical quality, the highest Verify3D score at 66.89%, and a very good ERRAT score of 94.90. Model 4 has a low QMEAN Z-Score of −5.64, good stereochemical quality, a Verify3D agreement of 56.29%, and a very good ERRAT score of 95.58. Model 5, with the lowest QMEAN Z-Score of −5.74, maintains good stereochemical quality, a Verify3D agreement of 54.30%, and an excellent ERRAT score of 97.96. Overall, **Model 1** stands out due to its balanced high QMEAN Z-Score, excellent stereochemical quality, and highest ERRAT score, making it the most reliable despite its moderate Verify3D percentage.

Table 7 highlights the quality assessments of five models. Model 1, with a QMEAN Z-Score of −3.94, 46.3% favored regions in the Ramachandran Plot, 36.93% Verify3D agreement, and a moderate ERRAT score of 73.6695, indicates low overall quality. Model 2, with a QMEAN Z-Score of −2.14, 71.3% favored regions, 63.61% Verify3D agreement, and an excellent ERRAT score of 89.2562, demonstrates high quality and reliability. Model 3 has a QMEAN Z-Score of −4.05, 47.9% favored regions, 43.67% Verify3D agreement, and a very good ERRAT score of 84.8485. Model 4, with the highest QMEAN Z-Score of −0.92, 60.7% favored regions, 43.67% Verify3D agreement, and an excellent ERRAT score of 87.6033, indicates the highest quality but moderate confidence. Model 5, with a QMEAN Z-Score of −2.02, 64.3% favored regions, 57.68% Verify3D agreement, and a very good ERRAT score of 84.8485, shows good overall quality. Thus, **Model 2** is the most reliable based on the balance of QMEAN Z-Score, Ramachandran Plot distribution, and ERRAT score.

### 3.10 Refinement of tertiary structure

The GalaxyRefine web server was employed to improve the structural fidelity and quality of the modeled protein. This process encompassed energy minimization and loop refinement, essential for attaining a high-resolution predicted structure. Initially, the “basic” vaccine model underwent refinement on the GalaxyRefine platform, resulting in the generation of five distinct model structures. The refinement process on GalaxyRefine is designed to optimize local geometry, particularly in loop regions, and to reduce steric clashes and unfavorable interactions within the protein structure, thereby enhancing the overall accuracy and reliability of the predicted models.

Among the structures developed in SM1, **Model 5** emerged as the most significant, demonstrating an RMSD of 0.598, a MolProbity score of 1.230, a clash score of 0.8, no poor rotamers, and a Rama favored value of 92.4%. In SM2, **Model 1** was distinguished by an RMSD of 0.408, a MolProbity score of 2.340, a clash score of 21.8, no poor rotamers, and a Rama favored value of 91.3%. For SM3, **Model 3** was the most notable, with an RMSD of 0.544, a MolProbity score of 2.546, a clash score of 27.4, a poor rotamer percentage of 1.1%, and a Rama favored value of 88.1%. These metrics indicate that these models are superior in structural quality and stability compared to the other generated models. The refined models of each vaccine construct were subsequently analyzed for molecular docking and dynamics study, with the results detailed in Supplementary Information, Tables S11, S12, and S13.

### 3.11 Molecular dynamics simulation of vaccine constructs

**Fig. 4:**
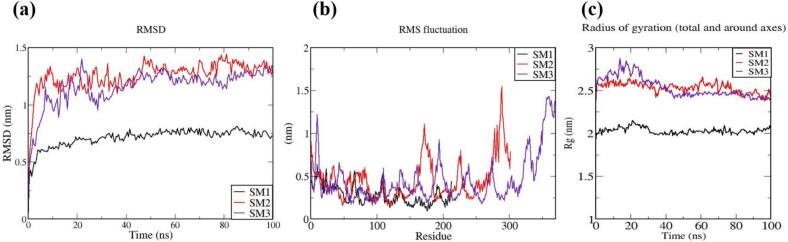
MD simulation trajectories for vaccine constructs SM1, SM2, and SM3 analyzed over a total duration of 100 ns. (a) RMSD plot for SM1, SM2 and SM3 (b) RMSF plot for SM1, SM2 and SM3 (c) Rg plot for SM1, SM2 and SM3.

Before conducting docking analysis, the three vaccine constructs were subjected to MD simulations to evaluate their stability, flexibility, and compactness. These assessments were carried out using RMSD, RMSF, and Rg plots. RMSD analysis revealed overall stability, with higher values indicating greater deviations from the initial structure, suggestive of potential instability. RMSF provided a detailed view of individual residue flexibility, highlighting regions with significant fluctuations that indicate areas of flexibility or rigidity. Rg analysis evaluated the compactness of the constructs, with variations in Rg values indicating changes in the molecule's overall shape and compactness during the simulation. The RMSD, RMSF and Rg plots of SM1, SM2, SM3 are shown in Fig. 4.

As illustrated in the RMSD simulation plot (Fig. 4a), the RMSD values for SM2 and SM3 exhibit a substantially greater increase compared to those of SM1. Throughout the simulation period, SM2 and SM3 show pronounced fluctuations in their RMSD values, indicating significant structural instability. In contrast, SM1 demonstrates remarkable stability, with its RMSD values remaining relatively constant and exhibiting minimal fluctuations, ranging from 0.73 to 0.81 nm after 80 ns. This suggests that the SM1 structure maintains a higher degree of stability over time compared to SM2 and SM3.

The RMSF plot (Fig. 4b) reveals that SM2 and SM3 exhibit higher residue- specific fluctuations compared to SM1. In SM2, the fluctuations range from 0.17 nm to 1.53 nm, while in SM3, the fluctuations range from 0.15 nm to 1.43 nm. Conversely, SM1 displays fluctuations ranging only from 0.1 nm to 0.63 nm. These findings indicate that SM1 has a relatively rigid structure, as evidenced by its lower range of fluctuations. In contrast, the higher fluctuation ranges observed in SM2 and SM3 suggest greater flexibility and dynamic movement of their residues.

The Rg value for SM1 was observed to be significantly lower, ranging from 1.91 nm to 2.13 nm, and remained nearly constant throughout the 100-nanosecond simulation, exhibiting minimal deviations. In contrast, the SM2 and SM3 constructs displayed greater variability in their Rg values, with deviations ranging from approximately 2.42 nm to 2.67 nm for SM2 and 2.39 nm to 2.90 nm for SM3, as illustrated in Fig. 4c. These findings indicate that the SM1 construct maintains increased compactness during its dynamic interactions compared to SM2 and SM3. The reduced fluctuations in the Rg values of SM1 suggest a more stable and tightly packed structure.

Taken together, SM1′s lower RMSD and RMSF values, coupled with its consistent Rg profile, indicate superior stability, rigidity, and compactness compared to SM2 and SM3. These attributes are critical for maintaining functional integrity under physiological conditions. Consequently, SM1 was prioritized for subsequent docking analyses, given its potential for enhanced performance and structural reliability.

### 3.12 Protein-protein docking

Protein-protein docking has become a key method for predicting interactions between immune receptors and vaccine constructs. To investigate the binding of the vaccine construct SM1 with immune receptors MHC-I, MHC-II, TLR2, and TLR4, molecular docking was performed using the ClusPro2.0 and HDOCK servers. The structures of MHC-I (PDB ID: 1I1Y), MHC-II (PDB ID: 1KG0), TLR2 (PDB ID: 6NIG), and TLR4 (PDB ID: 2Z62), obtained from the Protein Data Bank, had resolutions ranging from 1.70 Å to 2.65 Å. Before docking, the X-ray crystallography-derived structures of these receptors were processed in BIOVIA Discovery Studio Visualizer 2021 to remove crystallographic waters and co-crystallized molecules, retaining only the monomeric forms to ensure structural accuracy.

TLRs are critical for the innate immune response to bacterial infections. TLR2 identifies various bacterial components like lipoproteins and peptidoglycan, often partnering with TLR1 or TLR6 to recognize specific motifs. Activation of TLR2 initiates a signaling cascade that produces pro-inflammatory cytokines and antimicrobial peptides, and aids in the activation of antigen-presenting cells, enhancing adaptive immunity. TLR4, which can act synergistically with TLR2, responds to gram-positive infections by initiating the release of pro-inflammatory cytokines and chemokines, thereby driving inflammation, recruiting immune cells, and helping to eliminate pathogens.^120^, ^121^

MHC molecules play a pivotal role in the immune defense against bacterial infections by presenting antigens to T cells. MHC-I molecules present peptide fragments derived from endogenous antigens, including those from intracellular bacteria, on the surface of nearly all nucleated cells. This facilitates the recognition and elimination of infected cells by cytotoxic T lymphocytes, which is essential for managing intracellular bacterial infections. In contrast, MHC-II molecules, which are expressed on professional antigen-presenting cells (dendritic cells and macrophages), present exogenous antigens from extracellular bacteria that have been internalized and processed. Helper T cells detect these antigen-MHC-II complexes, leading to their activation and the coordination of a broad immune response. This includes the activation of B cells for antibody production and the enhancement of macrophage activity. Therefore, MHC-I and MHC-II together orchestrate a robust immune response against both intracellular and extracellular bacterial pathogens.^122^

ClusPro 2.0 and HDOCK each produced ten modeled complexes, from which the lowest energy complex was selected for analysis. Table 8 presents the docking results of the vaccine construct SM1 with four receptors (1I1Y, 1KG0, 2Z62, and 6NIG). For receptor 1I1Y, SM1 demonstrated a lowest energy of −716.3 kcal/mol using ClusPro and a docking energy of −266.29 kcal/mol using HDOCK, with a confidence score of 0.91, signifying a stable binding conformation. The 1KG0 receptor showed the lowest energy among all receptors in ClusPro at −848.1 kcal/mol and an HDock docking energy of −234.95 kcal/mol, with a confidence score of 0.85, suggesting reliable interactions. For the 2Z62 receptor, the ClusPro lowest energy was −768.7 kcal/mol and the HDock docking energy was −265.35 kcal/mol, with a confidence score of 0.91, indicating good binding stability. The 6NIG receptor exhibited the lowest ClusPro lowest energy at −897.1 kcal/mol and the lowest HDock docking energy at −269.14 kcal/mol, with the highest confidence score of 0.92, indicating the strongest binding interaction. Overall, the SM1 construct demonstrated strong and stable interactions with all tested receptors.

**Table 8:** Docking analysis of vaccine construct (SM1) with various immune receptors.

**Receptor**	**ClusPro**	**HDock**	
	**Lowest energy (kcal/mol)**	**Docking Energy (kcal/mol)**	**Confidence Score**
1I1Y	−716.3	−266.29	0.91
1KG0	−848.1	−234.95	0.85
2Z62	−768.7	−265.35	0.91
6NIG	−897.1	−269.14	0.92

A comprehensive examination of vaccine-immune receptor interactions was performed to elucidate the specific molecular mechanisms, including hydrogen bonding, hydrophobic effects, and electrostatic forces, that stabilize protein–protein complexes. The surrounding residues contributed to stability through a variety of non-covalent interactions such as hydrogen bonds, salt bridges, and additional non-bonded contacts. Interaction maps generated by PDBSum provided a detailed overview of the interactions between the SM1 vaccine construct and four receptors (1I1Y, 1KG0, 6NIG, and 2Z62).

**Fig. 5:**
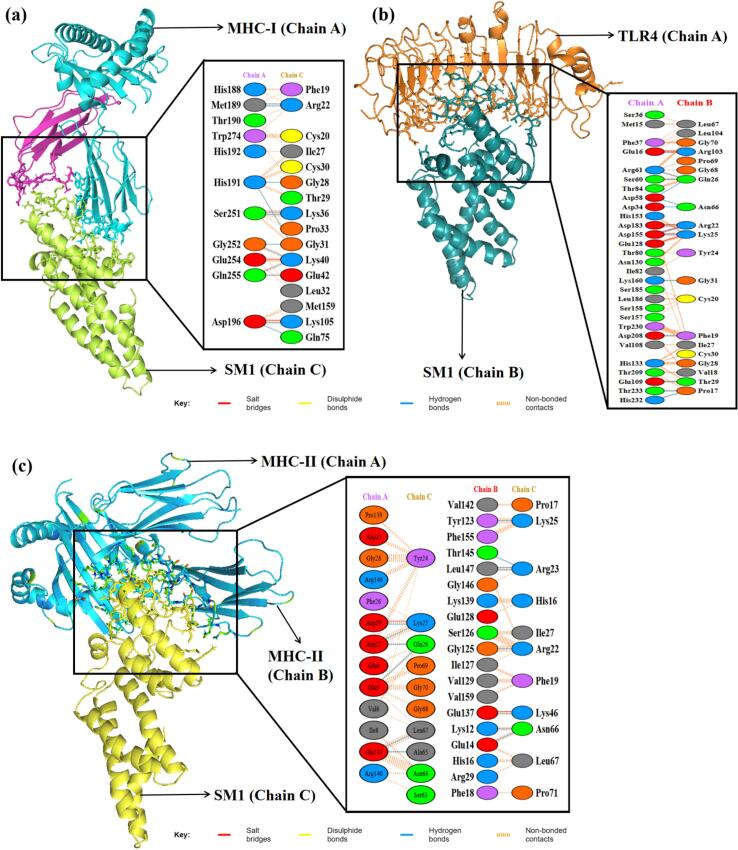

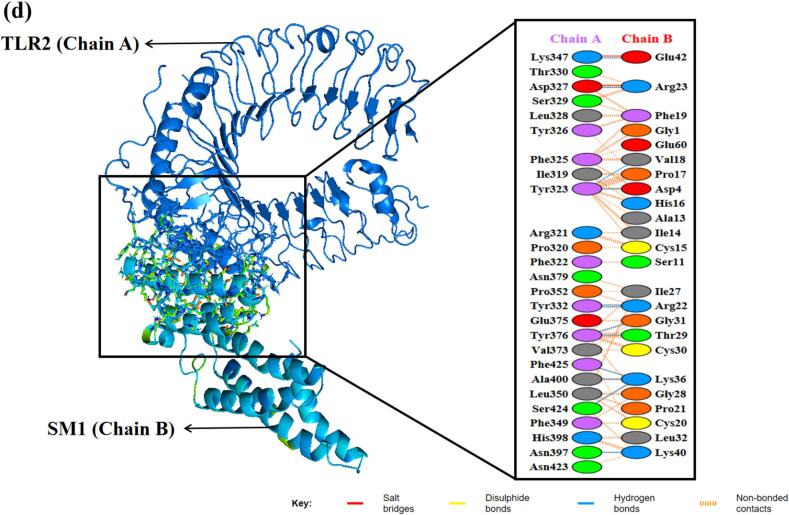
The molecular interactions of the vaccine candidate SM1 with membrane receptor binding sites. (a) Interaction with MHC class I, (b) binding to TLR4, (c) interaction with MHC class II, and (d) association with TLR2.

**Fig. 6:**
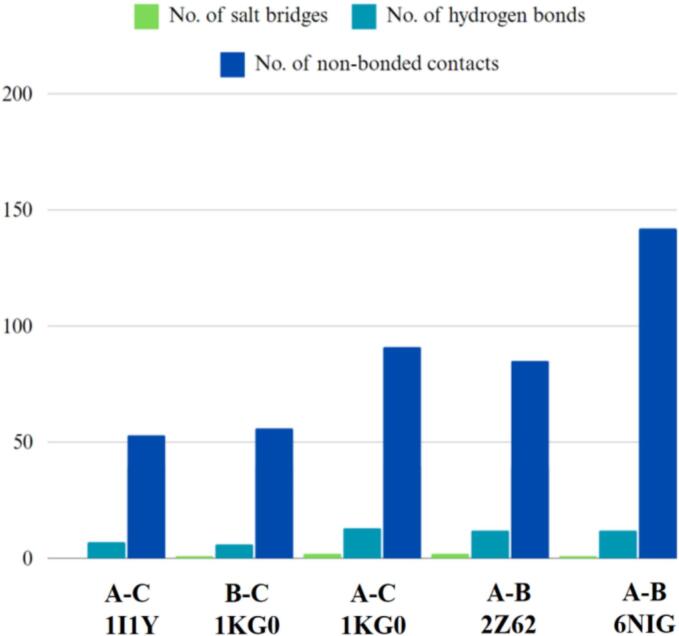
Bar chart depicting the total number of salt bridges, hydrogen bonds, and non-bonded interactions between the vaccine construct and immune receptors.

The analysis revealed that the 1I1Y receptor engaged with the A-C chain through 2 salt bridges, 12 hydrogen bonds, and 74 non-bonded contacts, indicating substantial stabilizing interactions. The 1KG0 receptor interacted with both A-C (1 salt bridge, 6 hydrogen bonds, 103 non-bonded contacts) and B-C chains (1 salt bridge, 8 hydrogen bonds, 76 non-bonded contacts), reflecting varied interface sizes and strong stabilizing interactions. The 2Z62 receptor showed interactions through A-B chains, including 5 salt bridges, 14 hydrogen bonds, and 147 non-bonded contacts, highlighting significant electrostatic and hydrophobic interactions. The 6NIG receptor demonstrated interactions with A-B chains, encompassing 2 salt bridges, 4 hydrogen bonds, and 190 non-bonded contacts, indicating extensive van der Waals interactions. In conclusion, SM1 demonstrated robust and varied interactions across these receptors, with particularly strong interactions observed with receptors 2Z62 and 6NIG, indicating stable and extensive binding configurations. The docked complexes along with the frequency of protein–protein stabilizing interactions of vaccine construct (SM1) with various immune receptors is illustrated in Fig. 5, Fig. 6 respectively.

### 3.13 Molecular dynamics simulation

MD simulations utilize an extensive model of interatomic interactions to forecast the dynamic behavior of individual atoms in proteins and other molecular systems. These simulations effectively capture critical biomolecular phenomena, including conformational transitions, ligand binding events, and protein folding dynamics. They offer detailed insights into the precise positions of all atoms with femtosecond temporal resolution.^123^

To evaluate the dynamics, stability, and immunogenicity of the SM1 vaccine construct, we performed 100-nanosecond MD simulations with MHC-I, MHC-II, TLR2, and TLR4. Post-simulation analyses employing RMSD, RMSF, and Rg metrics were conducted to elucidate the dynamic behavior and structural stability of these docking complexes under physiological conditions.

**RMSD: Root mean square deviation**

**Fig. 7:**
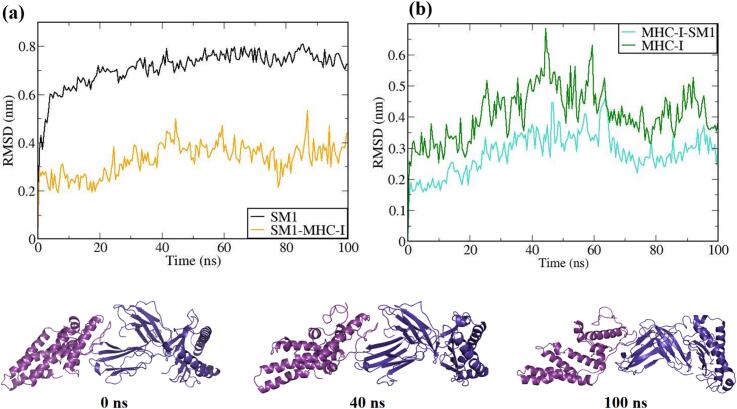
RMSD values for (a) SM1 both in its isolated state and in complex with MHC-I, and (b) MHC-I both in its isolated state and in complex with SM1.

**Fig. 8:**
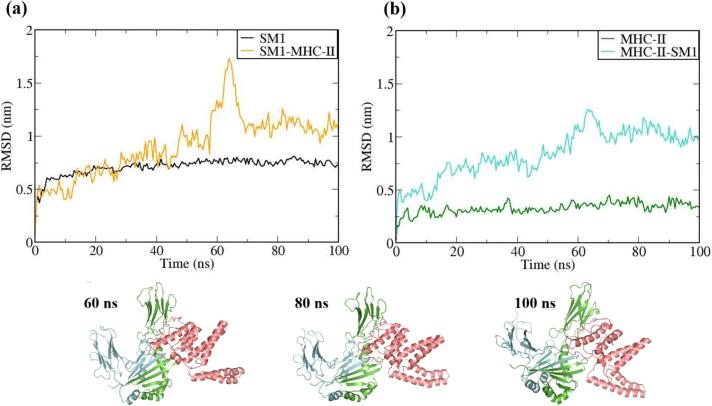
RMSD values for (a) SM1 in its isolated state and in complex with MHC-II, and (b) MHC-II in its isolated state and in complex with SM1.

**Fig. 9:**
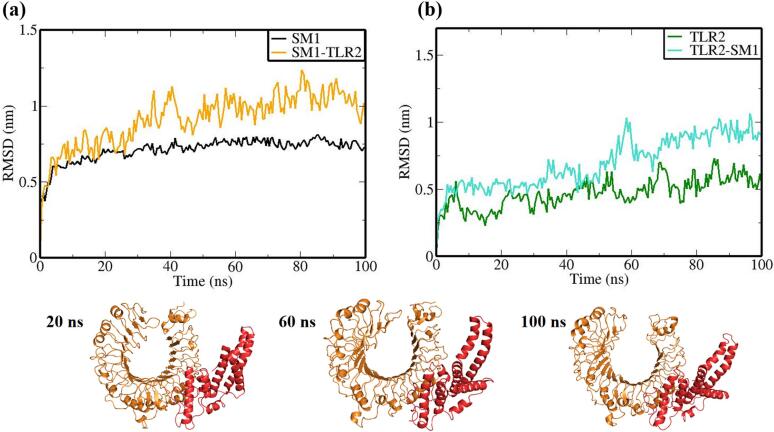
RMSD values for (a) SM1 in its isolated state and in complex with TLR2, and (b) TLR2 in its isolated state and in complex with SM1.

**Fig. 10:**
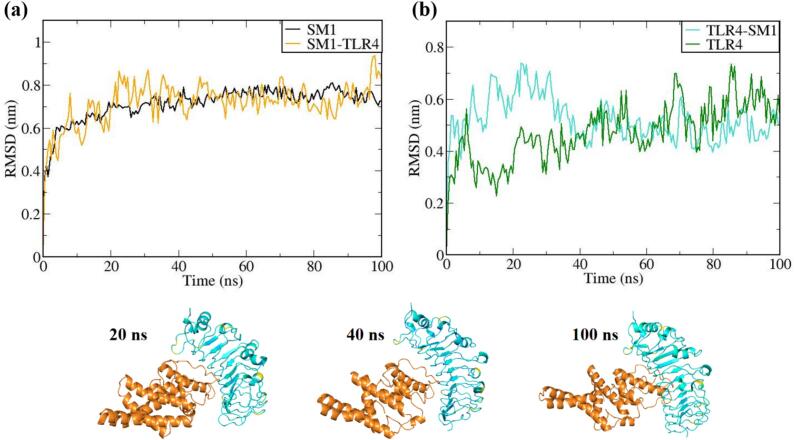
RMSD values for (a) SM1 in its isolated state and in complex with TLR4, and (b) TLR4 in its isolated state and in complex with SM1.

The RMSD plot is essential for assessing the equilibration of MD trajectories. The RMSD trajectory is a pivotal parameter for evaluating protein stability. RMSD backbone values were calculated over a 0 to 100 ns simulation period for the vaccine construct, immune receptor structures, and docked complexes. Higher RMSD values indicate reduced structural stability of the protein.^124^ Fig. 7, Fig. 8, Fig. 9, Fig. 10 depict the temporal evolution of the RMSD for the MEV construct, immune receptors, and MEV-immune receptor complexes. These figures also highlight the structural alterations observed in the key trajectories throughout the simulation period.

The RMSD plot in Fig. 7a demonstrates distinct stability profiles for SM1 and SM1 complexed with MHC-I. The RMSD of SM1 alone shows significant fluctuations up to approximately 40 ns, stabilizing between 0.68 nm and 0.8 nm after 45 ns, indicating initial conformational changes followed by stabilization. In contrast, the RMSD for the SM1-MHC-I complex exhibits significantly lower deviations, with fluctuations between 0.21 nm and 0.51 nm, indicating enhanced stability when bound to MHC-I. Fig. 7b shows the RMSD plot for MHC-I compared to the MHC-I-SM1 complex. The MHC-I-SM1 complex exhibits fluctuations with RMSD values ranging from 0.16 to 0.46 nm up to 67 ns, stabilizing thereafter with minor fluctuations between 0.21 to 0.36 nm. Conversely, MHC-I alone shows more significant fluctuations, with RMSD values ranging from 0.25 to 0.69 nm up to 65 ns, indicating greater conformational changes and reduced stability. After 65 ns, MHC-I tends to attain stabilization with RMSD values from 0.31 to 0.53 nm. Overall, these findings demonstrate that the SM1-MHC-I complex is more stable than SM1 and MHC-I, highlighting the stabilizing effect of SM1 on MHC-I.

From the RMSD plot of SM1 and SM1-MHC-II complex (Fig. 8a) it is observed that initially the fluctuations in both SM1 and SM1-MHC-II start with an RMSD around 0.4 nm. The RMSD for SM1 remains relatively low and stable throughout the simulation, fluctuating between 0.68 and 0.80 nm, suggesting high conformational stability. In contrast, the RMSD for the SM1-MHC-II complex increases significantly over time, peaking at around 1.7 nm by 64 ns, and then stabilizes between 0.85 and 1.25 nm post 70 ns, indicating substantial stabilizing conformational changes.

Fig. 8b illustrates the RMSD of the MHC-II protein and MHC-II-SM1 complex. Throughout the 100 ns simulation, the RMSD of the MHC-II protein remains relatively low, fluctuating between 0.24 and 0.45 nm, signifying sustained conformational stability. In contrast, the RMSD of the MHC-II-SM1 complex shows greater variability and higher values. The RMSD of the complex increases from 0.5 nm to approximately 1.25 nm by 63 ns and then stabilizes post 70 ns, fluctuating between 0.87 and 1.16 nm. These results indicate that the MHC-II protein maintains greater structural stability when not bound to SM1, while the binding of SM1 induces significant conformational changes in the MHC-II protein, resulting in higher and more variable RMSD values. This suggests that SM1 binding affects the structural integrity of the MHC-II protein, leading to increased flexibility or a shift to a different stable conformation. The enhanced flexibility likely arises from the reorganization of binding sites on the MHC-II molecule, which improves interactions with the vaccine construct and is crucial for efficient T cell signaling and activation.

Fig. 9a shows the RMSD of the SM1 construct alone, and SM1 in complex with the TLR2 receptor. Fluctuations in both SM1 and SM1-TLR2 complex start with an RMSD around 0.25 to 0.35 nm. The RMSD for SM1 increases slightly at the start but remains relatively low and stable throughout the simulation, fluctuating between 0.62 and 0.78 nm, indicating that the SM1 protein alone maintains a stable structure during the 100 ns simulation. In contrast, the RMSD for the SM1-TLR2 complex shows a higher degree of fluctuation and generally higher values compared to SM1 alone. The RMSD increases to around 0.9 nm within the first 31 ns and then fluctuates between 0.8 and 1.2 nm for the remainder of the simulation, suggesting significant conformational changes.

Fig. 9b illustrates the RMSD of the TLR2 protein alone and the TLR2-SM1 complex. Initially, both systems exhibit RMSD values between 0.3 and 0.4 nm, indicating stable conformations. For the TLR2 protein alone, RMSD values show a slight increase at the outset but remain relatively stable, fluctuating between 0.24 and 0.73 nm, reflecting consistent structural integrity throughout the simulation. Conversely, the TLR2-SM1 complex displays greater fluctuation and higher RMSD values, ranging from 0.48 to 1 nm up to 60 ns, suggesting substantial conformational changes. Beyond 60 ns, the RMSD of the TLR2-SM1 complex attempts to stabilize, fluctuating between 0.65 and 1.1 nm. This indicates that while the TLR2 protein alone maintains greater conformational stability, the TLR2-SM1 complex exhibits increased flexibility and adopts a distinct stable conformation due to the binding of the SM1 molecule. The increased flexibility suggests that TLR2 needs to undergo structural adjustments to effectively interact with SM1 to trigger an appropriate immune response.

The RMSD plot depicted in Fig. 10a demonstrates the temporal stability of molecular configurations for the SM1 and SM1-TLR4 systems. Initially, both the SM1 and SM1-TLR4 complexes exhibit minimal RMSD fluctuations, reflecting stability and similarity to their initial configurations. Over time, both systems experience increased RMSD variability, with the SM1-TLR4 complex displaying slightly higher fluctuations, indicative of greater structural changes or instability compared to SM1. Throughout the simulation, SM1′s RMSD varies between 0.59 nm and 0.8 nm. In contrast, the SM1-TLR4 complex shows initial RMSD fluctuations ranging from 0.45 nm to 0.89 nm up to 50 ns, after which it attempts to achieve a more stable conformation with fluctuations between 0.62 nm and 0.83 nm. However, towards the end of the simulation (97–100 ns), the RMSD for the SM1-TLR4 complex shows a slight increase, suggesting potential structural rearrangements or decreased stability. Overall, the lower and more stable RMSD for the SM1 system indicates it maintains structural integrity more effectively than the SM1-TLR4 complex.

The RMSD plot depicted in Fig. 10b demonstrates the stability of the TLR4-SM1 complex compared to the TLR4 receptor. Both systems exhibit low initial RMSD values, indicating initial stability and proximity to their starting configurations. However, as the simulation progresses, both systems show fluctuations. Notably, the TLR4 system displays greater RMSD increases than the TLR4-SM1 complex. Specifically, TLR4 experiences fluctuations ranging from 0.21 to 0.58 nm up to 40 ns, with subsequent stabilization at 0.39 to 0.71 nm post 40 ns. In contrast, the TLR4-SM1 complex shows higher initial fluctuations (0.38 to 0.72 nm) up to 40 ns but subsequently achieves a more stable conformation with reduced fluctuations (0.39 to 0.60 nm). Thus, the RMSD for TLR4 is consistently higher than that of the TLR4-SM1 complex, indicating superior stability of the TLR4-SM1 complex.

**RMSF: Root mean square fluctuation**

**Fig. 11:**
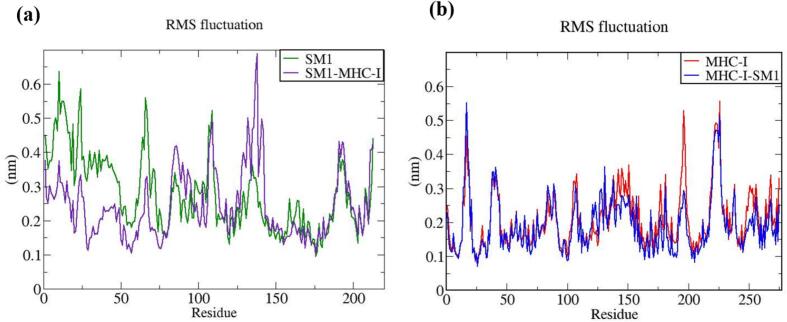
RMSF plot of the stretch with functionally important residues during the simulation period for (a) SM1 in its native state and SM1 in complex with MHC-I (b) MHC-I in its native state and MHC-I in complex with SM1.

**Fig. 12:**
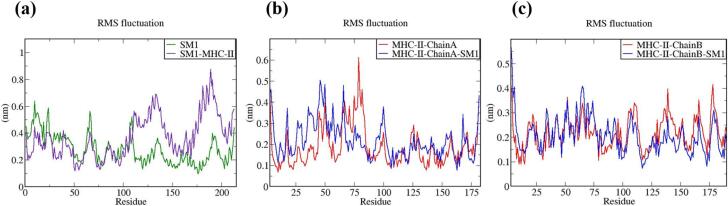
RMSF plot of the stretch with functionally important residues during the simulation period for (a) SM1 in its native state and SM1 in complex with MHC-II (b) Native MHC-II chain A and MHC-II chain A in complex with SM1. (c) Native MHC-II chain B and MHC-II chain B in complex with SM1.

**Fig. 13:**
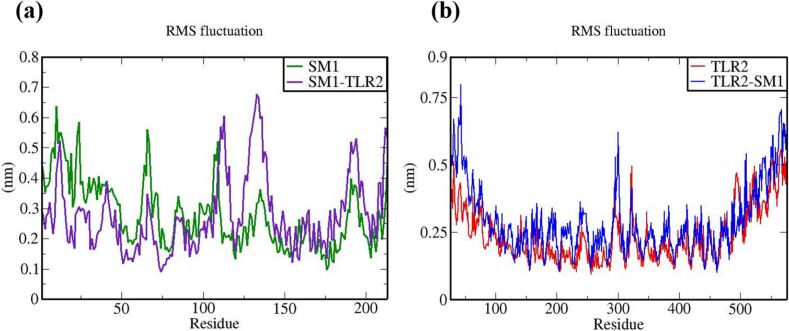
RMSF plot of the stretch with functionally important residues during the simulation period for (a) SM1 in its native state and SM1 in complex with TLR2 (b) TLR2 in its native state and TLR2 in complex with SM1.

**Fig. 14:**
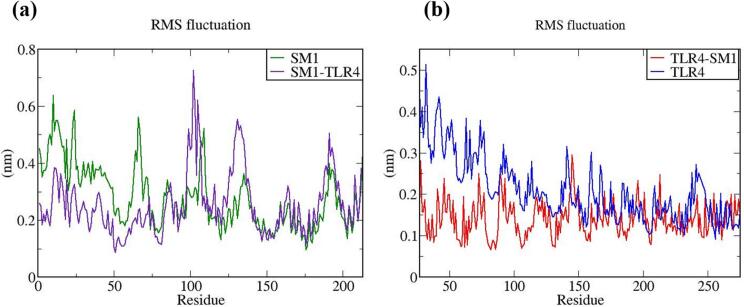
RMSF plot of the stretch with functionally important residues during the simulation period for (a) SM1 in its native state and SM1 in complex with TLR4 (b) TLR4 in its native state and TLR2 in complex with SM1.

To illustrate the variability in flexibility among individual residues, the RMSF is utilized with respect to the reference conformation from the standard MD simulation. Elevated RMSF values indicate that residues exhibit greater flexibility and dynamic movements relative to their average positions. Conversely, lower RMSF values suggest that residues have more restricted and limited displacements.^125^ Fig. 11, Fig. 12, Fig. 13, Fig. 14 show the RMSFs of the alpha carbon atoms for the vaccine construct, immune receptors, and all four docked complexes.

The RMSF plot in Fig. 11a assesses residue flexibility in the SM1 construct both isolated and in complex with MHC-I, elucidating the dynamic behavior during MD simulation. The RMSF of SM1, shows peaks at residues around 20, 25, 60, 110, and 210, with values up to ∼0.6 nm, indicating high flexibility regions. SM1 complexed with MHC-I, also exhibits peaks but generally lower, with significant peaks around residues 10, 25, 80, 110, and 190, reaching up to ∼0.5 nm, suggesting reduced flexibility however these residues do not fall in the binding region of SM1. Higher RMSF values for initial residues (1–75) in SM1 indicate greater flexibility, whereas the SM1-MHC-I complex shows reduced flexibility. Both states exhibit fluctuations in the middle residues (80–140), but SM1 has higher RMSF values, indicating more flexibility. For final residues (150–200), SM1 shows higher RMSF values, while the SM1-MHC-I complex shows lower values, indicating reduced flexibility. Overall, SM1 exhibits higher RMSF values, indicating greater flexibility, while the SM1-MHC-I complex shows reduced flexibility due to stabilizing interactions that constrain SM1 residue movement.

The RMSF plot for MHC-I and MHC-I-SM1, as shown in Fig. 11b, elucidates the flexibility and dynamic behavior of residues within these protein structures. Both proteins display a similar overall trend in RMS fluctuations, indicating comparable flexibility profiles. A comparative analysis reveals that MHC-I exhibits slightly higher peaks in the N-terminal region (residues 0–50) compared to MHC-I-SM1, suggesting that the SM1 variant enhances stability in this area. Between residues 50–150, MHC-I-SM1 exhibits slightly lower fluctuations, suggesting reduced flexibility. In contrast, residues 220–248 show higher peaks, indicating increased flexibility in this region. Notably, these residues are outside the MHC-II binding region, as demonstrated by the protein–protein interaction residues depicted in Fig. 5a. In the C-terminal region (200–275), MHC-I presents higher fluctuations around residues 159 and 225 compared to MHC-I-SM1, implying that the SM1 variant confers stabilization.

Fig. 12a depicts the RMSF of the systems SM1 and SM1-MHC-II complex. Both systems demonstrate variability in residue fluctuations, with RMSF values ranging from approximately 0.1 nm to slightly above 0.8 nm. Overall, the RMSF values for SM1-MHC-II are generally lower than those for SM1 alone, indicating reduced flexibility. However, certain regions, particularly around residues 110–140 and 175–200, exhibit higher RMSF values in the SM1-MHC-II system, suggesting areas of increased flexibility or dynamic behavior. While residues 50–105 show similar flexibility in both systems, notable differences are observed in the regions spanning residues 50–150 and 175–200, where SM1-MHC-II occasionally displays both elevated and reduced fluctuations compared to SM1. These findings imply that the presence of SM1 modulates the flexibility of specific regions in MHC-II, potentially influencing structural and functional aspects of their interaction.

Since chain A of the MHC class II receptor, in conjunction with chain B, presents antigenic peptides on professional antigen-presenting cells for recognition by the alpha–beta T-cell receptor on HLA-DR-restricted CD4+ T cells,^126^ RMSF analysis was conducted for both chain A and chain B of MHC-II, as shown in Fig. 12b and 12c, respectively.

Fig. 12b illustrates the RMSF for MHC-II-ChainA and MHC-II-ChainA-SM1, with values ranging from 0.05 nm to 0.6 nm, indicative of varying flexibility. Notably, from residues 0 to 70, MHC-II-ChainA-SM1 exhibits higher RMSF values, suggesting increased flexibility. The most significant fluctuation occurs around residues 70–80, where a prominent peak in MHC-II-ChainA is significantly reduced in the presence of SM1, indicating decreased flexibility and enhanced stabilization. From residues 110 to 175, both systems display similar trends, but MHC-II-ChainA consistently shows lower RMSF values, denoting reduced flexibility. Prominent peaks around residues 50 and 80 in MHC-II-ChainA alone indicate high flexibility, while regions around residues 110 and 150 exhibit relatively low RMSF values, reflecting stability in both systems.

Fig. 12c presents the RMSF profiles for MHC-II-ChainB and MHC-II-ChainB-SM1, with values spanning from approximately 0.05 nm to just over 0.5 nm, reflecting different degrees of residue flexibility. The overall flexibility of MHC-II-ChainB is minimally affected by the presence of SM1, as demonstrated by the close alignment of their RMSF profiles, with only slight deviations at particular residues. From residues 0–50, both systems exhibit comparable RMSF values, though MHC-II-ChainB alone shows marginally higher initial flexibility, indicating some stabilization by SM1. In the region from residues 55–97, SM1 binding slightly increases flexibility, as shown by higher RMSF values compared to MHC-II-ChainB alone, though these elevated fluctuations occur outside the binding region. Residues 125–175 exhibit similar RMSF profiles for both systems, with MHC-II-ChainB showing minor decreases in flexibility at specific points. Notably, residues 25–50 and 50–75 display relatively high RMSF values, with minor reductions in MHC-II-ChainB, suggesting slight stabilization, particularly around residue 65, where the MHC-II-ChainB-SM1 complex shows higher RMSF values, though these residues are outside the MHC-II binding region. Overall, the RMSF analysis indicates that SM1 binding to MHC-II-ChainB leads to minor flexibility variations, generally resulting in slight stabilization.

Fig. 13a presents the RMSF analysis of SM1 and the SM1-TLR2 complex. For residues 1–100, both SM1 and the SM1-TLR2 complex exhibit comparable fluctuation patterns, though SM1-TLR2 complex generally displays slightly reduced fluctuations. Between residues 100–150, a notable divergence is observed, with the SM1-TLR2 complex showing increased fluctuations relative to SM1. For residues 150–220, fluctuation patterns are similar between the two systems, but SM1-TLR2 tends to exhibit marginally higher fluctuations overall. Significant fluctuation peaks are evident in the SM1-TLR2 complex around residues 110–140 and 175–200, suggesting high flexibility that may correspond to loop or unstructured regions, however these residues are outside the SM1 binding region. Both SM1 and SM1-TLR2 show lower fluctuations in the 50–100 and 150–175 residue ranges, indicative of more rigid or structured regions.

Fig. 13b shows the RMSF analysis of TLR2 residues with and without SM1. Residues 0–100 exhibit high fluctuations in both TLR2 and the TLR2-SM1 complex, with the latter showing slightly higher peaks. In the 100–290 residue range, both cases display reduced fluctuations, indicating a more rigid domain; however, the TLR2-SM1 complex shows marginally increased fluctuations in certain areas outside the TLR2 binding region. For residues 350–500, primarily within the TLR2 binding region, the TLR2-SM1 complex demonstrates lower fluctuations, suggesting enhanced stability upon SM1 binding. The high fluctuation in residues 0–100 for both forms indicates a flexible N-terminal region, partially stabilized by SM1. The increased fluctuation in residues 500–550 upon SM1 binding suggests induced flexibility or conformational changes. Both TLR2 and TLR2-SM1 exhibit lower fluctuations in the 100–300 residue range, indicating a stable core domain that maintains rigidity despite SM1 binding.

The plot in Fig. 14a illustrates the RMSF of residues for SM1 alone and the SM1-TLR4 complex. This study examined RMSF values to understand the structural stability and flexibility changes upon complex formation. For residues 0–50, SM1 displayed RMSF values between 0.2 and 0.6 nm, indicating moderate fluctuation. In contrast, the SM1-TLR4 complex showed lower values (0.1 to 0.38 nm), suggesting reduced flexibility due to TLR4 binding. Residues 50–100 in SM1 had a significant peak around residue 70, reaching nearly 0.6 nm, whereas the SM1-TLR4 complex showed a reduced peak (∼0.24 nm), indicating stabilization of this flexible region upon TLR4 binding. For residues 100–150, SM1 exhibited RMSF values of 0.18 to 0.45 nm, with the SM1-TLR4 complex showing increased fluctuations (0.3 to 0.75 nm); however, these residues are not within the binding region. Residues 150–200 maintained consistent RMSF values (0.2 to 0.4 nm) in SM1, with minor peaks slightly lower in the SM1-TLR4 complex, indicating subtle structural rigidity upon complex formation. For residues 200–250, SM1 showed minor peaks (0.15 to 0.45 nm), while the SM1-TLR4 complex exhibited a notable increase in fluctuation, though these regions are outside the binding site. Overall, RMSF analysis indicates that TLR4 binding generally stabilizes the SM1 structure, with reduced RMSF values in most regions. Notably, residues 0–100 and 150–185 demonstrate significant stabilization, suggesting conformational adaptation upon TLR4 binding.

Fig. 14b presents the RMSF for TLR4 and the TLR4-SM1 complex. The TLR4-SM1 complex generally shows lower fluctuations compared to TLR4 alone, suggesting that SM1 binding stabilizes the TLR4 structure. While RMSF patterns are similar, there are significant differences in fluctuation amplitude. Notably, residues ∼0–125 show higher fluctuations in TLR4 (0.35–0.5 nm) compared to reduced fluctuations in TLR4-SM1 (around 0.06–0.3 nm), indicating substantial stabilization by SM1. Similarly, residues ∼125–250 exhibit peaks of about 0.3 nm in TLR4 and around 0.15–0.25 nm in TLR4-SM1. The reduced flexibility in the TLR4-SM1 complex implies that SM1 binding enhances TLR4 stabilization, potentially affecting its conformational dynamics and functional state. Regions with higher initial flexibility in TLR4 that show decreased fluctuations in the complex are likely crucial for the TLR4-SM1 interaction.

**Rg: Radius of gyration**

**Fig. 15:**
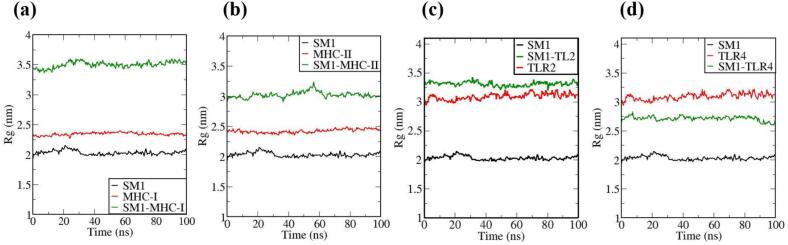
Radius of Gyration for various molecular systems over a simulation time of 100 ns (a) Rg for SM1, MHC-I, and the SM1-MHC-I complex (b) Rg for SM1, MHC-II, and the SM1-MHC-II complex (c) Rg for SM1, TLR2, and the SM1-TL2 complex (d) Rg for SM1, TLR4 and the SM1-TLR4 complex.

To assess the compactness of the vaccine construct, immune receptors, and the docked complex, the radius of gyration (Rg) was evaluated. Rg represents the root mean square distance of a set of atoms from their collective center of mass, serving as an indicator of the protein's structural compactness.^124^ An elevated Rg value indicates that protein folding is loose and the compactness is low, suggesting that the molecules are distributed farther from the center of mass.^127^ Fig. 15a, b, c, and d depict the temporal evolution of Rg values for the MEV construct, immune receptors, and MEV-immune receptor complexes, demonstrating that all components retain stability and compactness over the 100 ns simulation period.

Fig. 15a depicts the Rg over time (0–100 ns) for three systems: SM1, MHC-I, and the SM1-MHC-I complex. SM1′s Rg remains stable around 2.0 nm, indicating a compact, stable structure with minimal conformational changes. MHC-I shows Rg fluctuations around 2.26 nm, suggesting a slightly larger, more flexible structure with stable overall dimensions. The SM1-MHC-I complex exhibits the highest Rg, averaging around 3.5 nm, reflecting a significantly larger, more extended structure due to conformational rearrangements upon binding with SM1. The stable radius of gyration for SM1 and MHC-I indicates preserved structural integrity, whereas the increased Rg of the SM1-MHC-I complex underscores dynamic interactions and conformational changes essential for its immunological activity.

The Rg plot in Fig. 15b depicts the radius of gyration for SM1, MHC-II, and the SM1-MHC-II complex. SM1′s Rg remains consistently around 2.0 nm throughout the simulation, indicating a stable, compact structure with minimal conformational changes. This stability suggests that SM1 possesses a well-defined and rigid architecture with limited flexibility. In contrast, MHC-II exhibits fluctuations around 2.4 nm, suggesting a moderately larger and more flexible structure compared to SM1. Despite these fluctuations, MHC-II maintains a stable overall size, indicating internal flexibility while preserving its general dimensions. The SM1-MHC-II complex, however, shows a higher Rg, fluctuating around 3.0 nm, indicative of a significantly more extended and less compact structure than either SM1 or MHC-II. These fluctuations suggest dynamic interactions and potential conformational rearrangements within the complex, reflecting increased flexibility and expansion due to the interaction between SM1 and MHC-II. Thus, SM1 is the most compact structure with the lowest Rg, MHC-II is moderately compact with some flexibility, and the SM1-MHC-II complex is less compact, exhibiting a high Rg and considerable structural flexibility and expansion upon complex formation.

Fig. 15c presents the Rg for SM1, TLR2, and the SM1-TL2 complex. Throughout the simulation, SM1 maintains a stable Rg around 2.0 nm, reflecting minimal conformational changes and consistent compactness. Conversely, TLR2 shows a slightly higher Rg, with fluctuations ranging from 2.9 nm to 3.21 nm, averaging approximately at 3.0 nm, indicating a relatively extended conformation inherent to its structure. The Rg for SM1-TL2 complex begins at about 3.3 nm and stabilizes around 3.26 nm after 40 ns, suggesting reduced compactness due to binding dynamics with SM1. The Rg of the SM1-TL2 complex implies that, although less compact than SM1, it achieves a stability level akin to TLR2, suggesting that SM1 binding to TLR2 may foster a stable and compact structure.

Fig. 15d presents the Rg for SM1, TLR4, and the SM1-TLR4 complex. Throughout the simulation, SM1 maintains a stable Rg around 2.0 nm, indicating minimal conformational changes and consistent compactness. In contrast, TLR4 shows a higher average Rg of approximately 3.15 nm, suggesting reduced compactness due to its intrinsic flexibility. The SM1-TLR4 complex exhibits an average Rg ranging from 2.6 to 2.8 nm, implying that the binding interaction introduces flexibility, resulting in a stable yet less compact structure compared to SM1. These variations in Rg highlight the impact of molecular interactions on structural compactness, with SM1 being the most compact and TLR4 the least. The comparatively lower Rg of the SM1-TLR4 complex suggests that the binding interaction stabilizes the complex while allowing flexibility, potentially facilitating functional interactions in immunological contexts.

### 3.14 Post-simulation analysis of protein-protein stabilization interactions

Upon completing the 100 ns MD simulation of the vaccine-immune receptor complex, PDB files were extracted at various intervals from 0 to 100 ns. The final PDB structure at 100 ns was selected for analysis to identify the interacting residues and assess conformational changes in the vaccine construct within the equilibrated water box system's binding pocket of the immune receptors. PDBsum was utilized to plot the interacting residues.

**Fig. 16:**
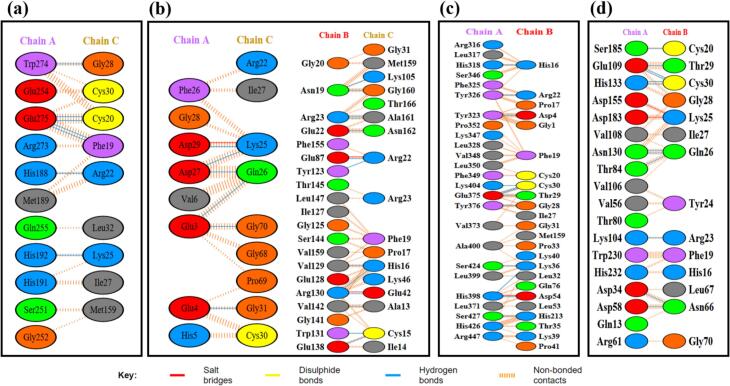
Residue-specific interaction diagram of SM1 with (a) MHC-I (where chain A denotes the receptor chain and chain C denotes the peptide chain), (b) MHC-II (where chains A and B denote the receptor chains and chain C denotes the peptide chain), (c) TLR-2 (where chain A denotes the receptor chain and chain B denotes the peptide chain), and (d) TLR-4 (where chain A denotes the receptor chain and chain B denotes the peptide chain).

The amino acid residues involved in interactions exhibit stabilization through the formation of diverse non-covalent interactions. These include hydrogen bonds (represented in blue), salt bridges (represented in red), and other non-bonded contacts (represented in orange), as depicted in Fig. 16.

Table 9 details the interactions between various receptor chains, specifying the number of interface residues, salt bridges, hydrogen bonds, and non-bonded contacts. For receptor 1I1Y (Chains A-C), Chain A and Chain C each have 11 and 9 interface residues, respectively. This interaction is characterized by the absence of salt bridges, 7 hydrogen bonds, and 53 non-bonded contacts, indicating a moderate interaction primarily stabilized by hydrogen bonds and van der Waals forces. For receptor 1KG0 (Chains A-C), Chains A and C have 8 and 9 interface residues, respectively, with 1 salt bridge, 6 hydrogen bonds, and 56 non-bonded contacts, suggesting a comparable interaction level to 1I1Y. In receptor 1KG0 (Chains B-C), Chains B and C exhibit 20 and 17 interface residues, respectively, with 2 salt bridges, 13 hydrogen bonds, and 91 non-bonded contacts. This configuration indicates a more extensive interaction interface with significant stabilization from ionic interactions, hydrogen bonds, and van der Waals forces.

**Table 9:** Protein–protein stabilizing interactions of vaccine construct with various immune receptors.

**Vaccine-Receptor Complex**	**Chain**	**Number of interface residues**	**Number of salt bridges**	**Number of hydrogen bonds**	**Number of non-bonded contacts**
**SM1-1I1Y**	A-C	11-9	0	7	53
**SM1-1KG0**	A-C	8-9	1	6	56
	B-C	20-17	2	13	91
**SM1-2Z62**	A-B	18-14	2	12	85
**SM1-6NIG**	A-B	25-24	1	12	142

*Chain C in 1I1Y and 1KG0 and chain B in 2Z62 and 6NIG denotes vaccine construct.

For receptor 2Z62 (Chains A-B), 18 and 14 interface residues are on Chains A and B, respectively, with 2 salt bridges, 12 hydrogen bonds, and 85 non-bonded contacts, highlighting substantial interaction stabilized by both ionic and van der Waals forces. The receptor 6NIG (Chains A-B) exhibits the most extensive interaction interface, with 25 and 24 interface residues on Chains A and B, respectively, 1 salt bridge, 12 hydrogen bonds, and 142 non-bonded contacts, reflecting the largest number of contacts and extensive reliance on van der Waals forces. Following MD simulations, the interactions between various receptor chains and the vaccine construct are predominantly stabilized by hydrogen bonds and non-covalent interactions, with salt bridges contributing minimally. The strength and stability of these interactions are correlated with the extent of interface residues and the frequency of non-covalent contacts, with 6NIG exhibiting the largest interaction surface.

### 3.15 Prediction of discontinuous B-cell epitopes

The protein folding process generates conformational B-cell epitopes. Utilizing the Ellipro tool from the IEDB server, five discontinuous B-cell epitopes were identified, with scores between 0.628 and 0.794. These epitopes vary in length from 3 to 47 residues. Comprehensive details about the amino acid residues, their quantities, and the corresponding scores for these epitopes are provided in SI Table S14.

### 3.16 Optimized codon usage and computational cloning of the final vaccine construct

Codon adaptation is a technique used to improve the translation efficiency of heterologous genes in a host organism, especially when codon usage differs between the donor and host species. In this study, the MEV gene was optimized for enhanced expression and immune response. The optimization was performed using the JCAT server to align MEV's codon usage with that of the *E. coli* K12 expression system, enabling high protein production.

The optimized SM1 construct, consisting of 639 base pairs, exhibited a GC content of 49.92%, which falls within the optimal range of 30–70% typically preferred for *E. coli* expression systems. This GC content contributes to genomic stability and promotes efficient transcription, thereby enhancing the construct's suitability for robust expression in *E. coli* K12. Additionally, the construct achieved a CAI of 1.0, reflecting a perfect match between the gene's codon usage and the host's preferred codon usage. This optimal CAI value ensures efficient translation and supports high levels of protein expression, further validating the construct's design for *E. coli*-based expression systems.

**Fig. 17:**
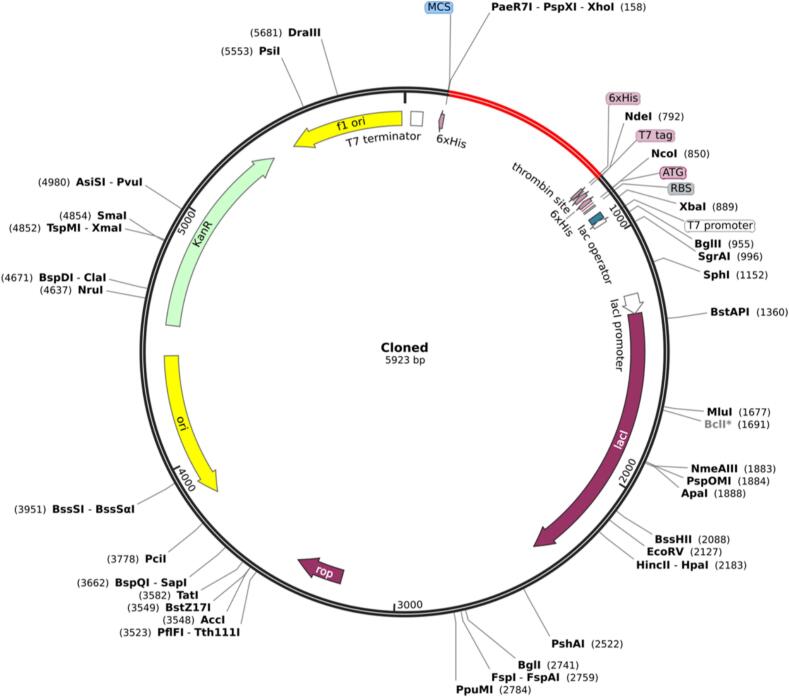
*In silico* cloning of the multi-epitope vaccine sequence (SM1) for restriction cloning into the pET28a(+) expression vector. In the diagram, the red line denotes the gene coding for the vaccine, while the black circle indicates the vector backbone. (For interpretation of the references to colour in this figure legend, the reader is referred to the web version of this article.)

To facilitate cloning, restriction sites for XhoI and NdeI were strategically added to the 5′ (N-terminal) and 3′ (C-terminal) ends of the optimized MEV sequence, respectively. These sites enhance the efficiency of ligation and cloning processes. The adapted MEV nucleotide sequence was then cloned into the *E. coli* pET28a(+) vector, verified using SnapGene 4.2. The final recombinant construct, encompassing the vector and the optimized codon sequence, totals 5923 base pairs. Fig. 17 provides an illustration of the cloned vaccine structure.

### 3.17 Immune simulation for vaccine efficacy

Vaccination represents a potent method for preventing bacterial infections by inducing strong immune responses, such as T-cell and B-cell activation, and memory cell formation. To assess the immune response elicited by the vaccine, we employed an *in silico* simulation approach using the C-ImmSim server. This tool models immune responses over time, offering insights into antigen-specific immune activity and the longevity of memory cell responses. This approach results in a subset of cells significantly increasing their lifespan, thereby outliving other cells. The immune simulation outcomes from the ImmSim server demonstrated alignment with real immune responses.

**Fig. 18:**
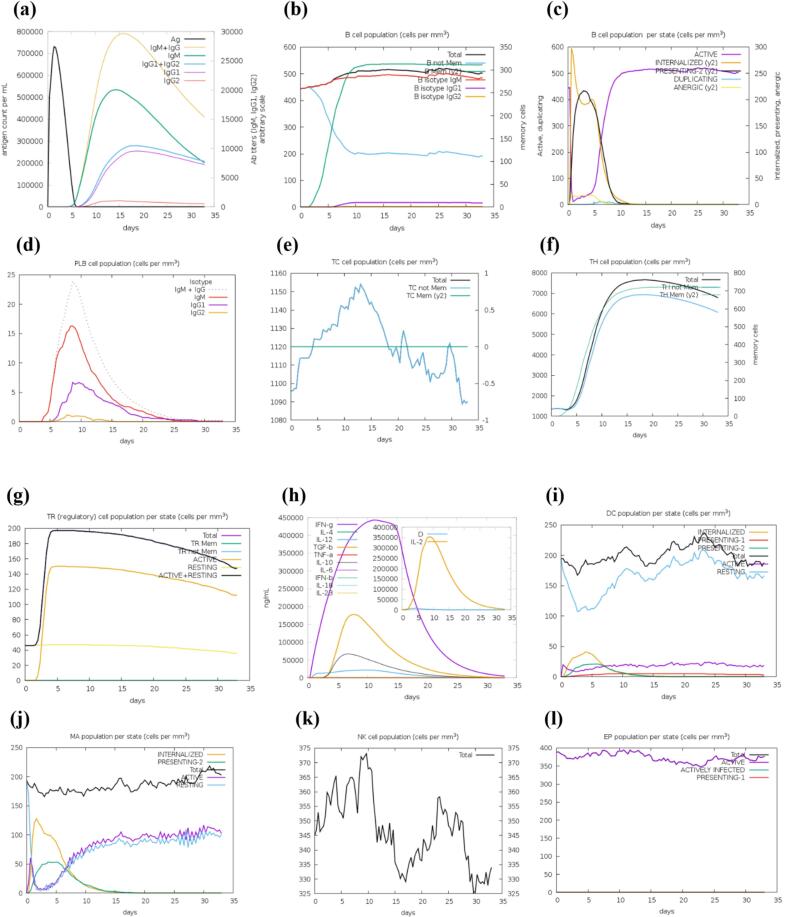
Immune response simulation of the SM1 vaccine candidate performed via the C-ImmSim server. (a) Production of immunoglobulins in response to antigen exposure, with distinct subclasses represented as colored peaks; (b) Enumeration of B-cell types involved in the immune response; (c) Expansion of B-cell populations across different states; (d) Plasma B lymphocyte counts categorized by isotype; (e) Generation of cytotoxic T-cell populations; (f) Generation of helper T-cells; (g) T regulatory cell levels; (h) Cytokine profiles post-injection, with an inset plot showing IL-2 levels and the Simpson index (D); (i) Dendritic cell populations per state; (j) Total macrophage counts; (k) Total natural killer cell counts. Cells in the resting state were not presented with the antigen, while those in the anergic state displayed antigen tolerance. (l) The generation and expansion of epithelial cell populations across various states.

Immune simulation analysis revealed that the vaccine construct induces robust primary and secondary immune responses, alongside the activation of the immune regulatory network. The primary response was marked by elevated IgM levels, while the secondary response was characterized by increased IgG levels, signifying the formation of immune memory. The antigen was cleared from the system within a week, while antibody titers remain elevated for an extended period, providing ongoing protection. An increase in B-cell populations was also noted, accompanied by heightened expression of immunoglobulins (IgG1, IgG2, IgM, and IgG + IgM), which contributed to a decrease in antigen levels. The B-cells exhibited extended survival and gave rise to memory B-cells. Upon re-exposure to the antigen, these memory B-cells facilitate robust and effective antibody-mediated immune responses (Fig. 18a, b, c, d).

Exposure to the vaccine construct led to a marked increase in helper T-cells (Th) and cytotoxic T-cells (Tc) cell populations, accompanied by the formation of memory cells. The cytotoxic T-cell count rose to 1155 cells per mm3 by days 15–16, eventually stabilizing at an optimal level of 1100 cells per mm^3^ by day 35. The T-helper cell count showed a gradual increase within 2–3 days post-vaccine administration, reaching levels of 7000–7500 cells/mm^3^ and maintaining this high concentration for the 35-day analysis period. The expansion of the T-cell population resulted in a significant increase in T-memory cells, which play a vital role in orchestrating immune responses and eliminating viral infections. These memory cells enable the immune system to swiftly and effectively respond to re-exposure to the antigen, promoting its clearance via established cytotoxic pathways. Additionally, exposure to SM1 led to a substantial rise in T-regulatory (Treg) cells, with levels reaching 110–150 cells per mm^3^. Tregs are crucial for regulating the immune response during bacterial infections. They maintain immune balance by suppressing excessive inflammation, preventing tissue damage, and ensuring a controlled response that clears bacteria without harming the host. Tregs also aid in resolving inflammation post-infection and preventing autoimmunity by protecting the body’s own tissues from erroneous attacks (Fig. 18e, f, g).

The vaccine elicited the production of key cytokines, including IFN-γ, IL-6, and IL-2, accompanied by a favorable Simpson Index (D), indicative of a broad and diverse immune response. An increase in (D) over time suggests the emergence of distinct epitope-specific T-cell clones. A lower (D) value reflects reduced diversity within B-cell populations post-vaccination. Cytokine levels peaked notably between days 10 and 15 following administration. The early and substantial elevations in IFN-γ, IL-6, and IL-2 suggest their crucial role in initiating a robust adaptive immune response, whereas the later peaks in TGF-β, IL-28, IL-10, and IL-18 indicate the onset of a regulatory phase aimed at modulating the immune response (Fig. 18h).

Vaccine administration induces a significant expansion of macrophages, dendritic cells, and natural killer (NK) cells, both in active and resting states, while preserving epithelial cell populations. This enhancement strengthens the immune response against bacterial infections (Fig. 18i, j, k, l). During the observation period, the proportion of active immune cells remained stable, with only a minimal presence of actively infected cells. These findings demonstrate that the vaccine robustly induces a comprehensive immune response, marked by sustained immune cell longevity, elevated immunoglobulin expression, and the activation of key cytokines such as IFN-γ and IL-2. The activation of these cytokines is crucial in enhancing the immune system's capacity to fight infections, thereby reinforcing the vaccine's efficacy.

While the *in silico* immune simulations presented in this study offer promising insights into the immunogenic potential of the designed vaccine construct, it is imperative to acknowledge the inherent limitations associated with immunoinformatics-based approaches. Computational models, although robust, are constrained by algorithmic assumptions and the quality of available training datasets, which may result in false-positive epitope predictions or insufficient representation of immunological heterogeneity across diverse human populations. Furthermore, despite comprehensive subtractive genomics filtering to eliminate homologous human sequences, the risk of immune cross-reactivity or off-target immune responses cannot be entirely excluded. Therefore, to substantiate the predictive findings and ensure translational relevance, it is essential that subsequent *in vitro* assays be conducted to evaluate antigen expression, structural integrity, and immunostimulatory properties, followed by *in vivo* studies in appropriate animal models to assess immunogenicity, safety, and protective efficacy under physiological conditions. These experimental validations will serve to confirm the computationally derived hypotheses and refine the vaccine candidate for potential clinical development.

Importantly, the limitations highlighted here do not diminish the utility of immunoinformatics; rather, they emphasize the need for an integrated workflow wherein computational predictions are iteratively aligned with empirical data. With ongoing advancements in algorithm design and expansion of high-resolution immunological databases, the accuracy, reliability, and translational applicability of immunoinformatics frameworks are expected to improve significantly, thereby enhancing their contribution to rational and accelerated vaccine development.

## 4 Conclusion

*Streptococcus pyogenes* is a major human pathogen known for causing a range of hemolytic diseases and chronic illnesses, with effective treatments remaining elusive, leading to severe, lifelong impacts. Developing therapeutic agents to combat these infections is crucial. Despite extensive vaccine development efforts against *Streptococcus pyogenes*, challenges such as reactogenicity have impeded progress and limited the number of vaccines tested.

Vaccination represents the most effective method for bolstering immune defenses against pathogens. However, developing effective live or attenuated vaccines is costly, time-consuming, and labor-intensive, with traditional vaccines often failing to stimulate a sufficient immune response and potentially causing allergic reactions. In contrast, multi-epitope-based vaccines (MEBSVs) present notable advantages over conventional vaccines, such as cost-efficiency, heightened safety, and superior efficacy. Several strategies are currently utilized to design and produce effective epitope-based vaccines.

In this study, subtractive genomics and reverse vaccinology were employed to develop a MEBSV against *Streptococcus pyogenes*. These methodologies focus on identifying non-homologous sequences and predicting antigenic epitopes to create vaccines capable of eliciting robust and specific immune responses. This approach addresses the limitations of traditional vaccines and aims to establish more effective and safer vaccination strategies against *S. pyogenes*.

Advanced subtractive genomics and *in silico* analyses were used to pinpoint potential vaccine targets. The proteomes of *Homo sapiens* and *S. pyogenes* were analyzed, revealing 136,194 and 1602 proteins, respectively. After excluding paralogous proteins, 1594 non-paralogous *S. pyogenes* proteins were assessed. BLASTp analysis against the human proteome identified 334 non-homologous proteins, reducing the risk of cross-reactivity and toxicity. TBLASTN searches in the DEG database identified 71 essential proteins, corresponding to 110 UniProt IDs. Subcellular localization analysis indicated that 86.67 % of these proteins were cytoplasmic and 13.33 % were plasma membrane proteins. Proteins such as atpF, dnaA, hrcA, and ezrA were prioritized for their immune accessibility and potential to induce neutralizing antibodies.

Extensive evaluations of antigenicity, allergenicity, and toxicity confirmed the viability of the vaccine candidates. For the atpF protein, six stable and hydrophilic linear B-cell epitopes were identified, along with CTL and HTL epitopes that cover 95.6% and 86.92% of the global population, respectively. Three multi-epitope vaccine candidates (SM1, SM2, and SM3) were engineered with varying adjuvant sequences. All constructs demonstrated antigenicity, non-allergenicity, non-toxicity, and stability. Their secondary and tertiary structures were predicted, refined, and validated. Molecular dynamics and protein–protein docking analyses indicated that the SM1 construct showed optimal stability and binding interactions with relevant immune receptors. The vaccine is anticipated to elicit robust cellular and humoral immune responses, including high levels of IFN-γ, IL-10, and IL-2, and increased IgG and IgM production. Codon optimization yielded a CAI of 1.0 and a GC content of 49.92%, enhancing expression in the *E. coli* system, with the pET28a(+) vector supporting *in silico* restriction cloning.

In conclusion, this study employed subtractive genomics, computational biology, and structural bioinformatics to identify and evaluate potential multi-epitope vaccine candidates targeting *Streptococcus pyogenes*. Among the constructs analyzed, the SM1 vaccine candidate demonstrated promising *in silico* performance, including stability, extensive population coverage, and the ability to elicit both cellular and humoral immune responses. Its strong and stable interactions with key immune receptors, particularly MHC-II and TLR4, highlight its compatibility with the extracellular and Gram-positive nature of *S. pyogenes*.

While the *in silico* findings of this study provide a valuable foundation for vaccine design, they should be interpreted with caution, considering the inherent limitations of computational approaches. Although *in silico* analyses offer predictive insights into antigenicity, stability, and immune interactions, they do not fully account for the complexities of host-pathogen interactions, immunogenic processing, or potential off-target effects in biological systems. Therefore, these findings should be viewed as an initial framework for guiding vaccine development rather than as definitive evidence of efficacy.

To establish the clinical potential of the SM1 vaccine construct, rigorous *in vitro* and *in vivo* validation is necessary. Experimental studies, including functional assays, immunogenicity evaluations in relevant models, and preclinical safety assessments, are essential to confirm its stability, immune activation potential, and protective efficacy. Additionally, further research is required to address critical challenges, such as ensuring broad-spectrum efficacy across diverse *S. pyogenes* serotypes and minimizing the risk of unintended immune responses.

Despite these limitations, this study highlights the promise of genomics-driven immunoinformatics as a strategic approach to vaccine development. By integrating computational predictions with experimental validation, such methodologies can accelerate the identification of novel vaccine candidates, ultimately contributing to global efforts to combat *S. pyogenes* infections and antibiotic resistance.

## CRediT authorship contribution statement

**Manisha Agarwal:** Writing – original draft, Methodology, Investigation, Formal analysis, Data curation. **Sanjeeb Handique:** Writing – review & editing, Visualization, Investigation, Formal analysis. **Sanchaita Rajkhowa:** Writing – review & editing, Supervision, Project administration, Funding acquisition, Data curation, Conceptualization. **Abhichandan Das:** Validation, Investigation, Formal analysis. **Debashis Panda:** Validation, Investigation, Formal analysis. **Sami A. Al-Hussain:** Validation, Project administration, Funding acquisition. **Magdi E.A. Zaki:** Writing – review & editing, Visualization, Validation, Supervision, Funding acquisition.

## Declaration of competing interest

The authors declare that they have no known competing financial interests or personal relationships that could have appeared to influence the work reported in this paper.

## Data Availability

All data generated and/or analysed during this study are included in this published article [and its supplementary information files].
