# Supplementary material for: Targeting Streptococcus pyogenes atpF protein for multi-epitope vaccine development: a genomics-driven immunoinformatics strategy
Source: J Genet Eng Biotechnol. 2025 Aug 5;23(3):100546. doi: 10.1016/j.jgeb.2025.100546 (PMC12375216; doi:10.1016/j.jgeb.2025.100546)
Supplement: Supplementary Data 1 [file mmc1.docx]

**Targeting Streptococcus pyogenes atpF Protein for Multi-Epitope Vaccine Development: A Genomics-Driven Immunoinformatics Strategy**

Manisha Agarwal^a^, Sanjeeb Handique^a^, Sanchaita Rajkhowa^a*^, Abhichandan Das^b^, Debashis Panda^b,c^ Sami A. Al-Hussain^d^, Magdi E. A. Zaki^d*^

^a^Centre for Biotechnology and Bioinformatics, Dibrugarh University, Dibrugarh-786004, Assam, India.

^b^Bioinformatics and Computational Biology Centre, DBT-APSCS&T CoE for Bioresources and Sustainable Development, Kimin-791121, Arunachal Pradesh, India.

^c^DBT-APSCS&T CoE for Bioresources and Sustainable Development, Kimin-791121, Arunachal Pradesh, India.

^d^Department of Chemistry, Imam Mohammad Ibn Saud Islamic University (IMSIU), Riyadh, Saudi Arabia.

^*^Correspondence: (Dr. Sanchaita Rajkhowa) [s_rajkhowa@dibru.ac.in](mailto:s_rajkhowa@dibru.ac.in) (ORCID ID: 0000-0002-4834-2654)

and (Dr. Magdi E. A. Zaki) [mezaki@imamu.edu.sa](mailto:mezaki@imamu.edu.sa)

**Table S1**. UniProt ID mapping results

| **Accession**  **Numbers** | **Protein names** | **Gene Names** |
| --- | --- | --- |
| WP_000529929.1 | Small ribosomal subunit protein uS3 (30S  ribosomal protein S3) | rpsC  SpyM50050 |
| WP_002986607.1 | DNA-directed RNA polymerase subunit alpha (RNAP subunit alpha) (EC 2.7.7.6) (RNA polymerase subunit alpha) (Transcriptase subunit  alpha) | rpoA SpyM50069 |
| WP_002988496.1 | Elongation factor P (EF-P) | efp  SpyM50303 |
| WP_002983310.1 | Heat-inducible transcription repressor HrcA | hrcA  SpyM50345 |
| WP_002995339.1 | UDP-N-acetylenolpyruvoylglucosamine reductase (EC 1.3.1.98) (UDP-N-acetylmuramate  dehydrogenase) | murB SpyM50963 |
| WP_002985152.1 | Translation initiation factor IF-3 | infC  SpyM51188 |
| WP_002985434.1 | Nucleotide-binding protein SpyM51325 | SpyM51325 |
| WP_002990580.1 | HPr kinase/phosphorylase (HPrK/P) (EC 2.7.11.-)  (EC 2.7.4.-) (HPr(Ser) kinase/phosphorylase) | hprK  SpyM51377 |
| WP_002985765.1 | Uridylate kinase (UK) (EC 2.7.4.22) (Uridine  monophosphate kinase) (UMP kinase) (UMPK) | pyrH  SpyM51489 |
| WP_002990948.1 | Probable manganese-dependent inorganic  pyrophosphatase (EC 3.6.1.1) (Pyrophosphate phospho-hydrolase) (PPase) | ppaC SpyM51539 |

| WP_002982092.1 | Small ribosomal subunit protein uS4 (30S  ribosomal protein S4) | rpsD  SpyM51805 |
| --- | --- | --- |
| WP_002990978.1 | Glutamate racemase (EC 5.1.1.3) | murI  Spy49_0297 |
| WP_002985434.1 | Nucleotide-binding protein Spy49_0545 | Spy49_0545 |
| WP_002990455.1 | Septation ring formation regulator EzrA | ezrA  Spy49_0561 |
| WP_002985152.1 | Translation initiation factor IF-3 | infC  Spy49_0629 |
| WP_002987659.1 | Chromosomal replication initiator protein DnaA | dnaA  spyM18_0001 |
| WP_002987659.1 | Chromosomal replication initiator protein DnaA | dnaA  SpyM3_0001 |
| WP_002987659.1 | Chromosomal replication initiator protein DnaA | dnaA SPs0001 |
| WP_002988496.1 | Elongation factor P (EF-P) | efp  SpyM3_1574 |
| WP_002988496.1 | Elongation factor P (EF-P) | efp SPs0293 |
| WP_002990455.1 | Septation ring formation regulator EzrA | ezrA  SpyM3_0477 |
| WP_002990455.1 | Septation ring formation regulator EzrA | ezrA SPs1377 |
| WP_011106808.1 | Phosphoglucosamine mutase (EC 5.4.2.10) | glmM  SpyM3_0671 |
| WP_011106808.1 | Phosphoglucosamine mutase (EC 5.4.2.10) | glmM SPs1182 |
| WP_010922169.1 | Isopentenyl-diphosphate delta-isomerase (IPP isomerase) (EC 5.3.3.2) (Isopentenyl diphosphate:dimethylallyl diphosphate isomerase) (Isopentenyl pyrophosphate isomerase) (Type 2  isopentenyl diphosphate isomerase) (IDI-2) | fni SpyM3_0598 |
| WP_010922169.1 | Isopentenyl-diphosphate delta-isomerase (IPP isomerase) (EC 5.3.3.2) (Isopentenyl  diphosphate:dimethylallyl diphosphate isomerase) | fni SPs1255 |

|  | (Isopentenyl pyrophosphate isomerase) (Type 2  isopentenyl diphosphate isomerase) (IDI-2) |  |
| --- | --- | --- |
| WP_002985152.1 | Translation initiation factor IF-3 | infC  SpyM3_0538 |
| WP_002985152.1 | Translation initiation factor IF-3 | infC SPs1316 |
| WP_002987881.1 | Transcription termination/antitermination protein  NusG | nusG  SpyM3_0127 |
| WP_002987881.1 | Transcription termination/antitermination protein  NusG | nusG SPs0129 |
| WP_002990948.1 | Probable manganese-dependent inorganic  pyrophosphatase (EC 3.6.1.1) (Pyrophosphate phospho-hydrolase) (PPase) | ppaC SpyM3_0278 |
| WP_002990948.1 | Probable manganese-dependent inorganic pyrophosphatase (EC 3.6.1.1) (Pyrophosphate  phospho-hydrolase) (PPase) | ppaC SPs1581 |
| WP_002985765.1 | Uridylate kinase (UK) (EC 2.7.4.22) (Uridine  monophosphate kinase) (UMP kinase) (UMPK) | pyrH  SpyM3_0326 |
| WP_002985765.1 | Uridylate kinase (UK) (EC 2.7.4.22) (Uridine  monophosphate kinase) (UMP kinase) (UMPK) | pyrH SPs1531 |
| WP_000529929.1 | Small ribosomal subunit protein uS3 (30S  ribosomal protein S3) | rpsC  SpyM3_0046 |
| WP_000529929.1 | Small ribosomal subunit protein uS3 (30S  ribosomal protein S3) | rpsC SPs0048 |
| WP_002982092.1 | Small ribosomal subunit protein uS4 (30S  ribosomal protein S4) | rpsD  SpyM3_1833 |
| WP_002982092.1 | Small ribosomal subunit protein uS4 (30S  ribosomal protein S4) | rpsD SPs1829 |
| WP_011106565.1 | tRNA(Ile)-lysidine synthase (EC 6.3.4.19) (tRNA(Ile)-2-lysyl-cytidine synthase)  (tRNA(Ile)-lysidine synthetase) | tilS SpyM3_0010 |
| WP_011106565.1 | tRNA(Ile)-lysidine synthase (EC 6.3.4.19) | tilS SPs0011 |

|  | (tRNA(Ile)-2-lysyl-cytidine synthase)  (tRNA(Ile)-lysidine synthetase) |  |
| --- | --- | --- |
| WP_002985053.1 | tRNA (guanine-N(1)-)-methyltransferase (EC 2.1.1.228) (M1G-methyltransferase) (tRNA  [GM37] methyltransferase) | trmD SpyM3_0574 |
| WP_002985053.1 | tRNA (guanine-N(1)-)-methyltransferase (EC 2.1.1.228) (M1G-methyltransferase) (tRNA  [GM37] methyltransferase) | trmD SPs1280 |
| WP_002985434.1 | Nucleotide-binding protein SpyM3_0462 | SpyM3_0462 |
| WP_002985434.1 | Nucleotide-binding protein SPs1393 | SPs1393 |
| WP_010922169.1 | Isopentenyl-diphosphate delta-isomerase (IPP isomerase) (EC 5.3.3.2) (Isopentenyl diphosphate:dimethylallyl diphosphate isomerase) (Isopentenyl pyrophosphate isomerase) (Type 2  isopentenyl diphosphate isomerase) (IDI-2) | fni spyM18_0940 |
| WP_002985152.1 | Translation initiation factor IF-3 | infC  spyM18_0866 |
| WP_002990948.1 | Probable manganese-dependent inorganic  pyrophosphatase (EC 3.6.1.1) (Pyrophosphate phospho-hydrolase) (PPase) | ppaC spyM18_0434 |
| WP_002985765.1 | Uridylate kinase (UK) (EC 2.7.4.22) (Uridine  monophosphate kinase) (UMP kinase) (UMPK) | pyrH  spyM18_0505 |
| WP_000529929.1 | Small ribosomal subunit protein uS3 (30S  ribosomal protein S3) | rpsC  spyM18_0057 |
| WP_000529929.1 | Small ribosomal subunit protein uS3 (30S  ribosomal protein S3) | rpsC gbs0064 |
| WP_000529929.1 | Small ribosomal subunit protein uS3 (30S  ribosomal protein S3) | rpsC SAG0064 |
| WP_002982092.1 | Small ribosomal subunit protein uS4 (30S  ribosomal protein S4) | rpsD  spyM18_2215 |
| WP_002986607.1 | DNA-directed RNA polymerase subunit alpha | rpoA |

|  | (RNAP subunit alpha) (EC 2.7.7.6) (RNA polymerase subunit alpha) (Transcriptase subunit  alpha) | spyM18_0079 |
| --- | --- | --- |
| WP_002988496.1 | Elongation factor P (EF-P) | efp  spyM18_1887 |
| WP_002987881.1 | Transcription termination/antitermination protein  NusG | nusG  spyM18_0159 |
| WP_002990580.1 | HPr kinase/phosphorylase (HPrK/P) (EC 2.7.11.-)  (EC 2.7.4.-) (HPr(Ser) kinase/phosphorylase) | hprK ptsK  spyM18_0653 |
| WP_002983310.1 | Heat-inducible transcription repressor HrcA | hrcA MGAS10750_  Spy1560 |
| WP_011184849.1 | Glycine--tRNA ligase alpha subunit (EC 6.1.1.14) (Glycyl-tRNA synthetase alpha subunit) (GlyRS) | glyQ MGAS10750_  Spy1496 |
| WP_002995339.1 | UDP-N-acetylenolpyruvoylglucosamine reductase (EC 1.3.1.98) (UDP-N-acetylmuramate  dehydrogenase) | murB MGAS10750_  Spy0976 |
| WP_002990420.1 | ATP synthase subunit b (ATP synthase F(0) sector subunit b) (ATPase subunit I) (F-type  ATPase subunit b) (F-ATPase subunit b) | atpF MGAS10750_  Spy0661 |
| WP_002985434.1 | Nucleotide-binding protein  MGAS10750_Spy0557 | MGAS10750_  Spy0557 |
| WP_002985765.1 | Uridylate kinase (UK) (EC 2.7.4.22) (Uridine monophosphate kinase) (UMP kinase) (UMPK) | pyrH MGAS10750_  Spy0378 |
| WP_002986607.1 | DNA-directed RNA polymerase subunit alpha (RNAP subunit alpha) (EC 2.7.7.6) (RNA polymerase subunit alpha) (Transcriptase subunit  alpha) | rpoA  MGAS10750_ Spy0074 |
| WP_000529929.1 | Small ribosomal subunit protein uS3 (30S | rpsC |

|  | ribosomal protein S3) | MGAS10750_  Spy0053 |
| --- | --- | --- |
| WP_002982092.1 | Small ribosomal subunit protein uS4 (30S ribosomal protein S4) | rpsD MGAS10270_  Spy1952 |
| WP_002988496.1 | Elongation factor P (EF-P) | efp MGAS10270_  Spy1614 |
| WP_002985152.1 | Translation initiation factor IF-3 | infC MGAS10270_  Spy0674 |
| WP_002985434.1 | Nucleotide-binding protein  MGAS10270_Spy0533 | MGAS10270_  Spy0533 |
| WP_002986607.1 | DNA-directed RNA polymerase subunit alpha (RNAP subunit alpha) (EC 2.7.7.6) (RNA polymerase subunit alpha) (Transcriptase subunit  alpha) | rpoA MGAS10270_ Spy0073 |
| WP_000529929.1 | Small ribosomal subunit protein uS3 (30S ribosomal protein S3) | rpsC MGAS10270_  Spy0052 |
| WP_002987659.1 | Chromosomal replication initiator protein DnaA | dnaA MGAS10270_  Spy0001 |
| WP_002982092.1 | Small ribosomal subunit protein uS4 (30S ribosomal protein S4) | rpsD MGAS9429_S  py1844 |
| WP_002988496.1 | Elongation factor P (EF-P) | efp MGAS9429_S  py1551 |
| WP_002985152.1 | Translation initiation factor IF-3 | infC  MGAS9429_S |

|  |  | py0674 |
| --- | --- | --- |
| WP_002990420.1 | ATP synthase subunit b (ATP synthase F(0) sector subunit b) (ATPase subunit I) (F-type  ATPase subunit b) (F-ATPase subunit b) | atpF MGAS9429_S  py0631 |
| WP_002990455.1 | Septation ring formation regulator EzrA | ezrA MGAS9429_S  py0607 |
| WP_002985765.1 | Uridylate kinase (UK) (EC 2.7.4.22) (Uridine monophosphate kinase) (UMP kinase) (UMPK) | pyrH MGAS9429_S  py0379 |
| WP_002990978.1 | Glutamate racemase (EC 5.1.1.3) | murI MGAS9429_S  py0302 |
| WP_002986607.1 | DNA-directed RNA polymerase subunit alpha (RNAP subunit alpha) (EC 2.7.7.6) (RNA polymerase subunit alpha) (Transcriptase subunit  alpha) | rpoA MGAS9429_S  py0070 |
| WP_000529929.1 | Small ribosomal subunit protein uS3 (30S ribosomal protein S3) | rpsC MGAS9429_S  py0050 |
| WP_002987659.1 | Chromosomal replication initiator protein DnaA | dnaA MGAS9429_S  py0001 |
| WP_000529929.1 | Small ribosomal subunit protein uS3 (30S  ribosomal protein S3) | rpsC  SAK_0097 |
| WP_002982092.1 | Small ribosomal subunit protein uS4 (30S  ribosomal protein S4) | rpsD  M28_Spy1865 |
| WP_002983310.1 | Heat-inducible transcription repressor HrcA | hrcA  M28_Spy1489 |
| WP_010922169.1 | Isopentenyl-diphosphate delta-isomerase (IPP  isomerase) (EC 5.3.3.2) (Isopentenyl  diphosphate:dimethylallyl diphosphate isomerase) (Isopentenyl pyrophosphate isomerase) (Type 2  isopentenyl diphosphate isomerase) (IDI-2) | fni  M28_Spy0665 |
| WP_002985053.1 | tRNA (guanine-N(1)-)-methyltransferase (EC 2.1.1.228) (M1G-methyltransferase) (tRNA  [GM37] methyltransferase) | trmD M28_Spy0637 |
| WP_002985152.1 | Translation initiation factor IF-3 | infC  M28_Spy0598 |
| WP_002990420.1 | ATP synthase subunit b (ATP synthase F(0) sector subunit b) (ATPase subunit I) (F-type  ATPase subunit b) (F-ATPase subunit b) | atpF M28_Spy0555 |
| WP_002990455.1 | Septation ring formation regulator EzrA | ezrA  M28_Spy0533 |
| WP_002985765.1 | Uridylate kinase (UK) (EC 2.7.4.22) (Uridine  monophosphate kinase) (UMP kinase) (UMPK) | pyrH  M28_Spy0365 |
| WP_002986607.1 | DNA-directed RNA polymerase subunit alpha (RNAP subunit alpha) (EC 2.7.7.6) (RNA polymerase subunit alpha) (Transcriptase subunit  alpha) | rpoA M28_Spy0069 |
| WP_000529929.1 | Small ribosomal subunit protein uS3 (30S  ribosomal protein S3) | rpsC  M28_Spy0049 |
| WP_002987659.1 | Chromosomal replication initiator protein DnaA | dnaA  M28_Spy0001 |

| WP_002982092.1 | Small ribosomal subunit protein uS4 (30S  ribosomal protein S4) | rpsD  M6_Spy1851 |
| --- | --- | --- |
| WP_002988496.1 | Elongation factor P (EF-P) | efp  M6_Spy1536 |
| WP_002983310.1 | Heat-inducible transcription repressor HrcA | hrcA  M6_Spy1494 |
| WP_011184849.1 | Glycine--tRNA ligase alpha subunit (EC 6.1.1.14)  (Glycyl-tRNA synthetase alpha subunit) (GlyRS) | glyQ  M6_Spy1433 |
| WP_002995339.1 | UDP-N-acetylenolpyruvoylglucosamine reductase (EC 1.3.1.98) (UDP-N-acetylmuramate  dehydrogenase) | murB M6_Spy0823 |
| WP_010922169.1 | Isopentenyl-diphosphate delta-isomerase (IPP isomerase) (EC 5.3.3.2) (Isopentenyl diphosphate:dimethylallyl diphosphate isomerase) (Isopentenyl pyrophosphate isomerase) (Type 2  isopentenyl diphosphate isomerase) (IDI-2) | fni M6_Spy0702 |
| WP_002985152.1 | Translation initiation factor IF-3 | infC  M6_Spy0636 |
| WP_002990580.1 | HPr kinase/phosphorylase (HPrK/P) (EC 2.7.11.-)  (EC 2.7.4.-) (HPr(Ser) kinase/phosphorylase) | hprK  M6_Spy0507 |
| WP_002985765.1 | Uridylate kinase (UK) (EC 2.7.4.22) (Uridine  monophosphate kinase) (UMP kinase) (UMPK) | pyrH  M6_Spy0404 |
| WP_002990948.1 | Probable manganese-dependent inorganic pyrophosphatase (EC 3.6.1.1) (Pyrophosphate  phospho-hydrolase) (PPase) | ppaC M6_Spy0345 |
| WP_002987881.1 | Transcription termination/antitermination protein  NusG | nusG  M6_Spy0185 |
| WP_002986607.1 | DNA-directed RNA polymerase subunit alpha (RNAP subunit alpha) (EC 2.7.7.6) (RNA polymerase subunit alpha) (Transcriptase subunit  alpha) | rpoA M6_Spy0119 |
| WP_000529929.1 | Small ribosomal subunit protein uS3 (30S  ribosomal protein S3) | rpsC  M6_Spy0099 |
| WP_002987659.1 | Chromosomal replication initiator protein DnaA | dnaA  M6_Spy0001 |
| WP_002984872.1 | Ribonuclease J 2 (RNase J 2) (EC 3.1.-.-) | rnj2  SpyM3_0657 |
| WP_002983310.1 | Heat-inducible transcription repressor HrcA | hrcA  spyM18_1833 |

**Table S2.** Predicted sub-cellular localization

| **Accession** | **GOterms** | **Score** |
| --- | --- | --- |
| sp_A2RBX3_DNAA_STR  PG | C:plasma membrane | 0.53 |
| sp_A2RC20_RS3_STRPG | C:cytoplasm | 0.70 |
| sp_A2RC40_RPOA_STRP  G | C:cytoplasm | 0.70 |
| sp_A2RCS2_EFP_STRPG | C:cytoplasm | 0.70 |
| sp_A2RCW4_HRCA_STR  PG | C:plasma membrane | 0.49 |

| sp_A2REL5_MURB_STRP  G | C:cytoplasm | 0.70 |
| --- | --- | --- |
| sp_A2RF84_IF3_STRPG | C:cytoplasm | 0.70 |
| sp_A2RFM4_Y1325_STRP  G | C:cytoplasm | 0.70 |
| sp_A2RFS6_HPRK_STRP  G | C:cytoplasm | 0.70 |
| sp_A2RG31_PYRH_STRP  G | C:cytoplasm | 0.70 |
| sp_A2RG81_PPAC_STRP  G | C:cytoplasm | 0.70 |
| sp_A2RGZ8_RS4_STRPG | C:cytoplasm | 0.70 |
| sp_B5XJX3_MURI_STRP  Z | C:cytoplasm | 0.70 |
| sp_B5XKL0_Y545_STRPZ | C:cytoplasm | 0.70 |
| sp_B5XKM5_EZRA_STR  PZ | C:plasma membrane | 0.92 |
| sp_B5XKT8_IF3_STRPZ | C:cytoplasm | 0.70 |
| sp_P0DB38_GLMM_STRP  3 | C:cytoplasm | 0.70 |
| sp_P0DB80_IDI2_STRP3 | C:cytoplasm | 0.70 |
| sp_P0DC78_NUSG_STRP  3 | C:cytoplasm | 0.70 |
| sp_P0DD14_PPAC_STRP3 | C:cytoplasm | 0.70 |
| sp_P0DD72_PYRH_STRP  3 | C:cytoplasm | 0.70 |
| sp_P0DG00_TILS_STRP3 | C:cytoplasm | 0.70 |
| sp_P0DG20_TRMD_STRP  3 | C:cytoplasm | 0.70 |
| sp_P0DG76_Y462_STRP3 | C:cytoplasm | 0.70 |

| sp_P0DG77_Y462_STRPQ | C:cytoplasm | 0.70 |
| --- | --- | --- |
| sp_Q1J5E0_SYGA_STRPF | C:cytoplasm | 0.70 |
| sp_Q1J7G3_ATPF_STRPF | C:plasma membrane | 0.87 |
| sp_Q1J7K4_Y557_STRPF | C:cytoplasm | 0.70 |
| sp_Q1JHS5_Y533_STRPD | C:cytoplasm | 0.70 |
| sp_Q8K7S6_RNJ2_STRP3 | C:cytoplasm | 0.70 |

**Table S3.** Antigenicity, Toxicity, and Allergenicity Profiles of Selected Proteins

| **Gene Name** | **Antigenicity**  **Score** | **Toxicity** | **Allerginicity** |
| --- | --- | --- | --- |
| dnaA | 0.643495 | Non-Toxin | Non- Allergen |
| hrcA | 0.261506 | Non-Toxin | Non- Allergen |
| ezrA | 0.770638 | Non-Toxin | Non- Allergen |
| atpF | 0.927767 | Non-Toxin | Non- Allergen |

**Table S4.** Ranking of B-Cell Epitopes Predicted by ABCpred Based on Scores

| **Rank** | **Sequence** | **Start**  **position** | **Score** |
| --- | --- | --- | --- |
| 1 | SKIISDAKEIGQLQGD | 78 | 0.87 |
| 2 | AEKIMGANLDKTAQSQ | 137 | 0.84 |
| 3 | SDAISAVKTEMSDLTV | 119 | 0.83 |
| 4 | SRDIDQAEQSRLSAQQ | 46 | 0.81 |
| 5 | DKLVAEATDEAKRLKE | 93 | 0.8 |
| 6 | SRLSAQQLEAKSQANL | 55 | 0.77 |
| 6 | TRSQQISRDIDQAEQS | 40 | 0.77 |
| 7 | DIEQSKSDAISAVKTE | 113 | 0.73 |
| 8 | WGAIESILQTRSQQIS | 31 | 0.72 |
| 9 | AKRLKEKALTDIEQSK | 103 | 0.71 |
| 10 | DKTAQSQLIDSYLDDL | 146 | 0.68 |
| 11 | MSITFGELVGNFILVT | 1 | 0.66 |
| 12 | AKSQANLDASRSQASK | 64 | 0.64 |
| 13 | VKTEMSDLTVLLAEKI | 125 | 0.6 |

**Table S5.** B-Cell Epitopes Predicted by IEDB

| **Position** | **Residue** | **Score** | **Assignment** |
| --- | --- | --- | --- |
| 0 | M | 0.231 | . |
| 1 | S | 0.283 | . |
| 2 | I | 0.349 | . |

| 3 | T | 0.411 | . |
| --- | --- | --- | --- |
| 4 | F | 0.477 | . |
| 5 | G | 0.498 | . |
| 6 | E | 0.52 | E |
| 7 | L | 0.515 | E |
| 8 | V | 0.525 | E |
| 9 | G | 0.514 | E |
| 10 | N | 0.492 | . |
| 11 | F | 0.46 | . |
| 12 | I | 0.44 | . |
| 13 | L | 0.415 | . |
| 14 | V | 0.395 | . |
| 15 | T | 0.384 | . |
| 16 | G | 0.369 | . |
| 17 | S | 0.341 | . |
| 18 | V | 0.335 | . |
| 19 | I | 0.347 | . |
| 20 | V | 0.353 | . |
| 21 | L | 0.348 | . |
| 22 | L | 0.356 | . |
| 23 | L | 0.364 | . |
| 24 | L | 0.375 | . |
| 25 | I | 0.392 | . |
| 26 | K | 0.421 | . |
| 27 | K | 0.435 | . |
| 28 | F | 0.445 | . |
| 29 | A | 0.439 | . |
| 30 | W | 0.468 | . |
| 31 | G | 0.467 | . |
| 32 | A | 0.47 | . |

| 33 | I | 0.449 | . |
| --- | --- | --- | --- |
| 34 | E | 0.459 | . |
| 35 | S | 0.458 | . |
| 36 | I | 0.472 | . |
| 37 | L | 0.473 | . |
| 38 | Q | 0.499 | . |
| 39 | T | 0.495 | . |
| 40 | R | 0.5 | E |
| 41 | S | 0.505 | E |
| 42 | Q | 0.527 | E |
| 43 | Q | 0.518 | E |
| 44 | I | 0.52 | E |
| 45 | S | 0.524 | E |
| 46 | R | 0.531 | E |
| 47 | D | 0.516 | E |
| 48 | I | 0.515 | E |
| 49 | D | 0.511 | E |
| 50 | Q | 0.509 | E |
| 51 | A | 0.504 | E |
| 52 | E | 0.511 | E |
| 53 | Q | 0.511 | E |
| 54 | S | 0.483 | . |
| 55 | R | 0.483 | . |
| 56 | L | 0.495 | . |
| 57 | S | 0.489 | . |
| 58 | A | 0.493 | . |
| 59 | Q | 0.49 | . |
| 60 | Q | 0.495 | . |
| 61 | L | 0.482 | . |
| 62 | E | 0.492 | . |

| 63 | A | 0.499 | . |
| --- | --- | --- | --- |
| 64 | K | 0.499 | . |
| 65 | S | 0.49 | . |
| 66 | Q | 0.502 | E |
| 67 | A | 0.497 | . |
| 68 | N | 0.487 | . |
| 69 | L | 0.487 | . |
| 70 | D | 0.494 | . |
| 71 | A | 0.483 | . |
| 72 | S | 0.477 | . |
| 73 | R | 0.48 | . |
| 74 | S | 0.488 | . |
| 75 | Q | 0.476 | . |
| 76 | A | 0.485 | . |
| 77 | S | 0.5 | . |
| 78 | K | 0.493 | . |
| 79 | I | 0.481 | . |
| 80 | I | 0.487 | . |
| 81 | S | 0.502 | E |
| 82 | D | 0.503 | E |
| 83 | A | 0.49 | . |
| 84 | K | 0.494 | . |
| 85 | E | 0.495 | . |
| 86 | I | 0.493 | . |
| 87 | G | 0.491 | . |
| 88 | Q | 0.517 | E |
| 89 | L | 0.525 | E |
| 90 | Q | 0.515 | E |
| 91 | G | 0.514 | E |
| 92 | D | 0.521 | E |

| 93 | K | 0.512 | E |
| --- | --- | --- | --- |
| 94 | L | 0.49 | E |
| 95 | V | 0.495 | E |
| 96 | A | 0.502 | E |
| 97 | E | 0.484 | . |
| 98 | A | 0.465 | . |
| 99 | T | 0.487 | . |
| 100 | D | 0.482 | . |
| 101 | E | 0.492 | . |
| 102 | A | 0.496 | . |
| 103 | K | 0.533 | E |
| 104 | R | 0.526 | E |
| 105 | L | 0.514 | E |
| 106 | K | 0.533 | E |
| 107 | E | 0.535 | E |
| 108 | K | 0.518 | E |
| 109 | A | 0.499 | E |
| 110 | L | 0.513 | E |
| 111 | T | 0.508 | E |
| 112 | D | 0.493 | . |
| 113 | I | 0.497 | . |
| 114 | E | 0.514 | E |
| 115 | Q | 0.492 | . |
| 116 | S | 0.484 | . |
| 117 | K | 0.496 | . |
| 118 | S | 0.504 | E |
| 119 | D | 0.478 | . |
| 120 | A | 0.452 | . |
| 121 | I | 0.458 | . |
| 122 | S | 0.451 | . |

| 123 | A | 0.441 | . |
| --- | --- | --- | --- |
| 124 | V | 0.433 | . |
| 125 | K | 0.443 | . |
| 126 | T | 0.438 | . |
| 127 | E | 0.454 | . |
| 128 | M | 0.447 | . |
| 129 | S | 0.477 | . |
| 130 | D | 0.472 | . |
| 131 | L | 0.466 | . |
| 132 | T | 0.44 | . |
| 133 | V | 0.457 | . |
| 134 | L | 0.463 | . |
| 135 | L | 0.449 | . |
| 136 | A | 0.42 | . |
| 137 | E | 0.442 | . |
| 138 | K | 0.442 | . |
| 139 | I | 0.434 | . |
| 140 | M | 0.43 | . |
| 141 | G | 0.47 | . |
| 142 | A | 0.479 | . |
| 143 | N | 0.493 | . |
| 144 | L | 0.514 | E |
| 145 | D | 0.534 | E |
| 146 | K | 0.533 | E |
| 147 | T | 0.532 | E |
| 148 | A | 0.539 | E |
| 149 | Q | 0.551 | E |
| 150 | S | 0.552 | E |
| 151 | Q | 0.529 | E |
| 152 | L | 0.514 | E |
| 153 | I | 0.492 | E |
| 154 | D | 0.502 | E |
| 155 | S | 0.5 | E |
| 156 | Y | 0.491 | . |
| 157 | L | 0.491 | . |
| 158 | D | 0.484 | . |
| 159 | D | 0.472 | . |
| 160 | L | 0.426 | . |
| 161 | G | 0.372 | . |
| 162 | E | 0.328 | . |
| 163 | A | 0.263 | . |

**Table S6.** HLA Alleles and Peptide Binding Characteristics of CTL (Cytotoxic T-Lymphocyte) as Predicted by IEDB Server

| **Allele** | **seq_num** | **start** | **end** | **length** | **peptide** | **IC50** | **rank** |
| --- | --- | --- | --- | --- | --- | --- | --- |
| HLA-A*02:03 | 3 | 15 | 23 | 9 | LLAEKIMGA | 2.82 | 0.03 |
| HLA-B*40:01 | 1 | 6 | 14 | 9 | GELVGNFIL | 3.1 | 0.01 |
| HLA-B*40:01 | 3 | 7 | 15 | 9 | TEMSDLTVL | 7.68 | 0.02 |
| HLA-A*02:03 | 3 | 14 | 23 | 10 | VLLAEKIMGA | 9.99 | 0.15 |
| HLA-A*01:01 | 3 | 9 | 17 | 9 | MSDLTVLLA | 10.05 | 0.02 |
| HLA-A*68:02 | 1 | 1 | 9 | 9 | MSITFGELV | 10.11 | 0.09 |
| HLA-A*02:06 | 1 | 29 | 37 | 9 | FAWGAIESI | 11.32 | 0.11 |
| HLA-A*02:01 | 3 | 15 | 23 | 9 | LLAEKIMGA | 11.78 | 0.1 |
| HLA-A*02:06 | 3 | 15 | 23 | 9 | LLAEKIMGA | 13.82 | 0.14 |
| HLA-A*68:02 | 1 | 7 | 15 | 9 | ELVGNFILV | 16.07 | 0.15 |
| HLA-B*40:01 | 1 | 5 | 14 | 10 | FGELVGNFIL | 16.18 | 0.04 |
| HLA-A*02:03 | 1 | 12 | 21 | 10 | FILVTGSVIV | 16.45 | 0.27 |
| HLA-A*02:03 | 1 | 13 | 21 | 9 | ILVTGSVIV | 17.45 | 0.29 |
| HLA-A*11:01 | 1 | 18 | 27 | 10 | SVIVLLLLIK | 23.73 | 0.13 |

| HLA-A*11:01 | 3 | 19 | 27 | 9 | KIMGANLDK | 24.39 | 0.13 |
| --- | --- | --- | --- | --- | --- | --- | --- |
| HLA-B*44:02 | 3 | 7 | 16 | 10 | TEMSDLTVLL | 25.54 | 0.05 |
| HLA-A*30:01 | 3 | 19 | 27 | 9 | KIMGANLDK | 27.64 | 0.14 |
| HLA-B*15:01 | 3 | 29 | 37 | 9 | AQSQLIDSY | 28.12 | 0.14 |
| HLA-A*02:03 | 2 | 45 | 54 | 10 | RLKEKALTDI | 28.19 | 0.46 |
| HLA-A*11:01 | 1 | 20 | 28 | 9 | IVLLLLIKK | 33.8 | 0.19 |
| HLA-A*02:06 | 2 | 35 | 43 | 9 | LVAEATDEA | 35.62 | 0.38 |
| HLA-A*68:02 | 3 | 8 | 16 | 9 | EMSDLTVLL | 36.74 | 0.31 |
| HLA-A*68:02 | 2 | 35 | 43 | 9 | LVAEATDEA | 37.81 | 0.31 |
| HLA-A*02:06 | 1 | 7 | 15 | 9 | ELVGNFILV | 38.98 | 0.41 |
| HLA-A*30:01 | 2 | 17 | 25 | 9 | ASKIISDAK | 41.68 | 0.2 |
| HLA-B*15:01 | 3 | 28 | 37 | 10 | TAQSQLIDSY | 42.91 | 0.23 |
| HLA-A*02:03 | 1 | 24 | 33 | 10 | LLIKKFAWGA | 43.76 | 0.68 |
| HLA-A*68:02 | 1 | 29 | 37 | 9 | FAWGAIESI | 45.64 | 0.35 |
| HLA-A*02:06 | 1 | 12 | 21 | 10 | FILVTGSVIV | 48.56 | 0.49 |
| HLA-A*02:03 | 3 | 15 | 24 | 10 | LLAEKIMGAN | 49.03 | 0.75 |
| HLA-B*40:01 | 1 | 6 | 15 | 10 | GELVGNFILV | 51.35 | 0.13 |
| HLA-A*31:01 | 2 | 5 | 14 | 10 | KSQANLDASR | 57.02 | 0.61 |
| HLA-A*02:06 | 1 | 13 | 21 | 9 | ILVTGSVIV | 57.86 | 0.57 |
| HLA-A*02:06 | 1 | 24 | 33 | 10 | LLIKKFAWGA | 58.97 | 0.58 |
| HLA-A*68:01 | 2 | 11 | 19 | 9 | DASRSQASK | 64.68 | 0.54 |
| HLA-B*40:01 | 3 | 7 | 16 | 10 | TEMSDLTVLL | 65.93 | 0.15 |
| HLA-A*03:01 | 3 | 19 | 27 | 9 | KIMGANLDK | 67.38 | 0.26 |
| HLA-A*02:01 | 3 | 14 | 23 | 10 | VLLAEKIMGA | 67.71 | 0.61 |
| HLA-A*30:01 | 2 | 12 | 20 | 9 | ASRSQASKI | 68.07 | 0.26 |
| HLA-B*40:01 | 3 | 6 | 15 | 10 | KTEMSDLTVL | 71.47 | 0.15 |
| HLA-A*02:06 | 1 | 6 | 15 | 10 | GELVGNFILV | 72.45 | 0.66 |
| HLA-A*02:01 | 1 | 29 | 37 | 9 | FAWGAIESI | 73.82 | 0.68 |
| HLA-A*02:03 | 1 | 6 | 15 | 10 | GELVGNFILV | 77.65 | 1 |
| HLA-A*02:01 | 1 | 13 | 21 | 9 | ILVTGSVIV | 79.38 | 0.71 |

| HLA-A*02:01 | 1 | 12 | 21 | 10 | FILVTGSVIV | 79.54 | 0.71 |
| --- | --- | --- | --- | --- | --- | --- | --- |
| HLA-A*31:01 | 1 | 39 | 47 | 9 | QTRSQQISR | 87.06 | 0.88 |
| HLA-A*02:01 | 1 | 24 | 33 | 10 | LLIKKFAWGA | 87.4 | 0.77 |
| HLA-A*11:01 | 1 | 19 | 27 | 9 | VIVLLLLIK | 89.83 | 0.55 |
| HLA-A*02:06 | 1 | 18 | 26 | 9 | SVIVLLLLI | 90.5 | 0.79 |
| HLA-A*02:03 | 3 | 7 | 16 | 10 | TEMSDLTVLL | 95.02 | 1.3 |
| HLA-A*01:01 | 3 | 8 | 17 | 10 | EMSDLTVLLA | 96.74 | 0.16 |
| HLA-A*68:01 | 2 | 16 | 25 | 10 | QASKIISDAK | 98.13 | 0.74 |
| HLA-B*40:01 | 2 | 2 | 10 | 9 | LEAKSQANL | 98.55 | 0.19 |
| HLA-A*02:06 | 1 | 1 | 9 | 9 | MSITFGELV | 100.26 | 0.85 |

**Table S7.** HLA Alleles and Peptide Binding Characteristics of HTL (Helper T-Lymphocyte) as Predicted by IEDB Server

| **Allele** | **seq_nu**  **m** | **star**  **t** | **en**  **d** | **lengt**  **h** | **core_peptid**  **e** | **peptide** | **scor**  **e** | **ran**  **k** |
| --- | --- | --- | --- | --- | --- | --- | --- | --- |
| HLA-  DRB1*07:01 | 1 | 34 | 48 | 15 | ILQTRSQQ  I | IESILQTRSQQISR  D | 0.97  7 | 0.0  3 |
| HLA-  DRB1*03:01 | 2 | 17 | 31 | 15 | IISDAKEIG | ASKIISDAKEIGQL  Q | 0.98  4 | 0.0  3 |
| HLA-  DRB1*07:01 | 1 | 33 | 47 | 15 | ILQTRSQQ  I | AIESILQTRSQQIS  R | 0.96  7 | 0.0  4 |
| HLA-  DRB1*03:01 | 2 | 16 | 30 | 15 | IISDAKEIG | QASKIISDAKEIGQ  L | 0.98 | 0.0  4 |
| HLA-  DRB1*03:01 | 2 | 15 | 29 | 15 | IISDAKEIG | SQASKIISDAKEIG  Q | 0.97  6 | 0.0  5 |
| HLA-  DRB1*07:01 | 1 | 32 | 46 | 15 | ILQTRSQQ  I | GAIESILQTRSQQI  S | 0.94  9 | 0.0  6 |
| HLA-  DRB1*07:01 | 1 | 35 | 49 | 15 | ILQTRSQQ  I | ESILQTRSQQISRD  I | 0.96  2 | 0.0  6 |
| HLA- | 2 | 18 | 32 | 15 | IISDAKEIG | SKIISDAKEIGQLQ | 0.96 | 0.0 |

| DRB1*03:01 |  |  |  |  |  | G | 8 | 7 |
| --- | --- | --- | --- | --- | --- | --- | --- | --- |
| HLA-  DRB1*04:01 | 2 | 32 | 46 | 15 | LVAEATDE  A | GDKLVAEATDEAK  RL | 0.91  8 | 0.1  7 |
| HLA-  DRB1*04:01 | 2 | 31 | 45 | 15 | LVAEATDE  A | QGDKLVAEATDEA  KR | 0.92  5 | 0.1  7 |
| HLA-  DRB1*03:01 | 2 | 14 | 28 | 15 | IISDAKEIG | RSQASKIISDAKEI  G | 0.89  8 | 0.3  4 |
| HLA-  DRB1*07:01 | 1 | 31 | 45 | 15 | ILQTRSQQ  I | WGAIESILQTRSQ  QI | 0.80  6 | 0.4  5 |
| HLA-  DRB1*04:01 | 2 | 30 | 44 | 15 | LVAEATDE  A | LQGDKLVAEATDE  AK | 0.81  3 | 0.6 |
| HLA-  DRB1*01:01 | 2 | 24 | 38 | 15 | IGQLQGD  KL | AKEIGQLQGDKLV  AE | 0.86  5 | 0.6 |
| HLA-  DRB1*03:01 | 2 | 19 | 33 | 15 | IISDAKEIG | KIISDAKEIGQLQG  D | 0.80  7 | 0.7  8 |
| HLA-  DRB1*15:01 | 1 | 34 | 48 | 15 | ILQTRSQQ  I | IESILQTRSQQISR  D | 0.69  5 | 0.9  5 |
| HLA-  DRB1*04:01 | 2 | 33 | 47 | 15 | LVAEATDE  A | DKLVAEATDEAKR  LK | 0.74  1 | 1.1 |
| HLA-  DRB1*01:01 | 2 | 23 | 37 | 15 | IGQLQGD  KL | DAKEIGQLQGDKL  VA | 0.78  1 | 1.2 |
| HLA-  DRB1*07:01 | 1 | 36 | 50 | 15 | ILQTRSQQ  I | SILQTRSQQISRDI  D | 0.65 | 1.3 |
| HLA-  DRB1*01:01 | 3 | 9 | 23 | 15 | LTVLLAE  KI | MSDLTVLLAEKIM  GA | 0.75  7 | 1.3 |
| HLA-  DRB1*15:01 | 1 | 33 | 47 | 15 | ILQTRSQQ  I | AIESILQTRSQQIS  R | 0.56  1 | 1.7 |
| HLA-  DRB1*01:01 | 3 | 8 | 22 | 15 | LTVLLAE  KI | EMSDLTVLLAEKI  MG | 0.64  5 | 2.1 |
| HLA-  DRB1*15:01 | 1 | 35 | 49 | 15 | ILQTRSQQ  I | ESILQTRSQQISRD  I | 0.48  2 | 2.3 |

| HLA-  DRB1*01:01 | 2 | 25 | 39 | 15 | IGQLQGD  KL | KEIGQLQGDKLVA  EA | 0.61  7 | 2.4 |
| --- | --- | --- | --- | --- | --- | --- | --- | --- |
| HLA-  DRB1*04:01 | 1 | 28 | 42 | 15 | WGAIESIL  Q | KFAWGAIESILQT  RS | 0.55  1 | 2.5 |
| HLA-  DRB1*03:01 | 1 | 42 | 56 | 15 | ISRDIDQA  E | SQQISRDIDQAEQ  SR | 0.59  8 | 2.6 |
| HLA-  DRB1*11:01 | 2 | 17 | 31 | 15 | IISDAKEIG | ASKIISDAKEIGQL  Q | 0.58 | 2.6 |
| HLA-  DRB1*01:01 | 3 | 10 | 24 | 15 | LTVLLAE  KI | SDLTVLLAEKIMG  AN | 0.55  2 | 2.9 |
| HLA-  DRB1*01:01 | 1 | 34 | 48 | 15 | ILQTRSQQ  I | IESILQTRSQQISR  D | 0.52  3 | 3.1 |
| HLA-  DRB1*01:01 | 2 | 22 | 36 | 15 | IGQLQGD  KL | SDAKEIGQLQGDK  LV | 0.52  6 | 3.1 |

**Table S8.** HLA Alleles and Peptide Binding Characteristics of HTL (Helper T-Lymphocyte) as Predicted by ProPred-II Server

| **Rank** | **Sequence** | **At Position** | **Score** | **% of Highest Score** |
| --- | --- | --- | --- | --- |
| **DRB1_0101** | | | | |
| 1 | FILVTGSVI | 11 | 2.25 | 37.5 |
| 2 | FGELVGNFI | 4 | 0.7 | 11.67 |
| 3 | LTVLLAEKI | 131 | 0.5 | 8.33 |
| 4 | LVTGSVIVL | 13 | 0.4 | 6.67 |
| **DRB1_0301** | | | | |
| 1 | IISDAKEIG | 79 | 5.1 | 53.68 |
| 2 | LVTGSVIVL | 13 | 4.16 | 43.79 |
| 3 | VLLAEKIMG | 133 | 4.1 | 43.16 |
| 4 | LQGDKLVAE | 89 | 3.8 | 40 |

| 5 | IVLLLLIKK | 19 | 3.5 | 36.84 |
| --- | --- | --- | --- | --- |
| 6 | LLLIKKFAW | 22 | 3.3 | 34.74 |
| 7 | LVGNFILVT | 7 | 2.9 | 30.53 |
| 8 | VLLLLIKKF | 20 | 2.7 | 28.42 |
| 9 | LLLLIKKFA | 21 | 2.4 | 25.26 |
| **DRB1_0401** | | | | |
| 1 | LVAEATDEA | 94 | 4.2 | 48.84 |
| 2 | WGAIESILQ | 30 | 2.88 | 33.49 |
| 3 | LLAEKIMGA | 134 | 2.2 | 25.58 |
| **DRB1_0701** | | | | |
| 1 | ILQTRSQQI | 36 | 6.5 | 56.03 |
| 2 | FILVTGSVI | 11 | 6.2 | 53.45 |
| 3 | ILVTGSVIV | 12 | 6.1 | 52.59 |
| 4 | LVTGSVIVL | 13 | 5.9 | 50.86 |
| 5 | VTGSVIVLL | 14 | 5.2 | 44.83 |
| 6 | MSITFGELV | 0 | 3.9 | 33.62 |
| 7 | LLLIKKFAW | 22 | 3.9 | 33.62 |
| 8 | WGAIESILQ | 30 | 3.7 | 31.9 |
| 9 | IEQSKSDAI | 113 | 3.3 | 28.45 |
| 10 | LTVLLAEKI | 131 | 3 | 25.86 |
| 11 | IKKFAWGAI | 25 | 2.8 | 24.14 |
| 12 | FGELVGNFI | 4 | 2.7 | 23.28 |
| 13 | FAWGAIESI | 28 | 2.7 | 23.28 |
| **DRB1_0801** | | | | |
| 1 | LLIKKFAWG | 23 | 2.7 | 31.4 |
| 2 | LLLLIKKFA | 21 | 2.2 | 25.58 |
| 3 | VLLAEKIMG | 133 | 1.8 | 20.93 |
| **DRB1_1101** | | | | |
| 1 | IVLLLLIKK | 19 | 3.9 | 46.99 |
| 2 | LLLLIKKFA | 21 | 3.2 | 38.55 |

| 3 | VIVLLLLIK | 18 | 3.1 | 37.35 |
| --- | --- | --- | --- | --- |
| 4 | WGAIESILQ | 30 | 2.6 | 31.33 |
| 5 | LVGNFILVT | 7 | 2.3 | 27.71 |
| 6 | VLLAEKIMG | 133 | 2.1 | 25.3 |
| 7 | LLLIKKFAW | 22 | 1.8 | 21.69 |
| **DRB1_1301** | | | | |
| 1 | VLLLLIKKF | 20 | 4 | 45.45 |
| 2 | LLLLIKKFA | 21 | 3.7 | 42.05 |
| 3 | VLLAEKIMG | 133 | 3.2 | 36.36 |
| 4 | LVGNFILVT | 7 | 3 | 34.09 |
| 5 | IVLLLLIKK | 19 | 3 | 34.09 |
| 6 | VIVLLLLIK | 18 | 2.4 | 27.27 |
| 7 | VGNFILVTG | 8 | 2.3 | 26.14 |
| 8 | LLLIKKFAW | 22 | 2.3 | 26.14 |
| 9 | LLIKKFAWG | 23 | 2.3 | 26.14 |
| 10 | LIKKFAWGA | 24 | 2.3 | 26.14 |
| 11 | LQTRSQQIS | 37 | 1.8 | 20.45 |
| **DRB1_1501** | | | | |
| 1 | LVGNFILVT | 7 | 3.85 | 39.29 |
| 2 | LVTGSVIVL | 13 | 3.8 | 38.78 |
| 3 | IKKFAWGAI | 25 | 3.8 | 38.78 |
| 4 | IVLLLLIKK | 19 | 3.6 | 36.73 |
| 5 | VGNFILVTG | 8 | 3.1 | 31.63 |
| 6 | FILVTGSVI | 11 | 2.8 | 28.57 |
| 7 | VIVLLLLIK | 18 | 2.5 | 25.51 |
| 8 | ILVTGSVIV | 12 | 2.4 | 24.49 |
| 9 | VLLAEKIMG | 133 | 2.4 | 24.49 |
| 10 | LLLIKKFAW | 22 | 2.3 | 23.47 |
| 11 | LLAEKIMGA | 134 | 2.15 | 21.94 |

**Table S9.** HLA Alleles and Peptide Binding Characteristics of HTL (Helper T-Lymphocyte) as Predicted by RANKPEP Server

| **RANK** | **PO S.** | **N- TERMINUS** | **SEQUENC E** | **C- TERMINUS** | **MW (Da)** | **SCOR E** | **% OPT.** |
| --- | --- | --- | --- | --- | --- | --- | --- |
| **DRB1s010 1** |  |  |  |  |  |  |  |
| 1 | 87 | AKE | IGQLQGDK  L | VAE | 953.1 | 15.772 | 32.69  % |
| 2 | 26 | LLL | IKKFAWGA  I | ESI | 992.26 | 12.727 | 26.38  % |
| 3 | 103 | TDE | AKRLKEK  AL | TDI | 1038.3 | 11.867 | 24.60  % |
| 4 | 31 | KFA | WGAIESIL  Q | TRS | 975.15 | 8.8 | 18.24  % |
| 5 | 70 | QAN | LDASRSQA  S | KII | 915.97 | 8.506 | 17.63  % |
| **DRB1s030**  **1c** |  |  |  |  |  |  |  |
| 1 | 80 | ASK | IISDAKEIG | QLQ | 927.07 | 21.733 | 53.37  % |
| 2 | 45 | SQQ | ISRDIDQA  E | QSR | 1028.1 | 19.398 | 47.63  % |
| **DRB1s040**  **1** |  |  |  |  |  |  |  |
| 1 | 95 | GDK | LVAEATDE  A | KRL | 899.96 | 13.536 | 30.70  % |
| 2 | 64 | QLE | AKSQANL  DA | SRS | 898.97 | 7.624 | 17.29  % |
| 3 | 31 | KFA | WGAIESIL | TRS | 975.15 | 6.826 | 15.48 |

|  |  |  | Q |  |  |  | % |
| --- | --- | --- | --- | --- | --- | --- | --- |
| 4 | 84 | ISD | AKEIGQLQ  G | DKL | 925.05 | 6.609 | 14.99  % |
| 5 | 143 | IMG | ANLDKTA  QS | QLI | 928.99 | 5.429 | 12.31  % |
| 6 | 30 | KKF | AWGAIESI  L | QTR | 918.1 | 5.422 | 12.30  % |
| 7 | 58 | SRL | SAQQLEA  KS | QAN | 943.03 | 5.239 | 11.88  % |
| **DRB1s070**  **1** |  |  |  |  |  |  |  |
| 1 | 124 | AIS | AVKTEMS  DL | TVL | 975.12 | 16.558 | 32.20  % |

**Table S10.** Characteristics of the constructed vaccines against *S.pyogenes*

| **Vaccine/**  **Adjuvant** | **SM1 (Beta-Defensin)** | **SM2 (50S ribosomal L7/L12 protein)** | **SM3 (HBHA Protein)** |
| --- | --- | --- | --- |
| **Allergenicity** | Non-allergen | Non-allergen | Non-allergen |
| **Toxicity** | Non-Toxin | Non-Toxin | Non-Toxin |
| **Antigenicity** | 0.8850 (Antigen) | 0.7217 (Antigen) | 0.7801 (Antigen) |

| **Length of amino acid** | 213 | 302 | 371 |
| --- | --- | --- | --- |
| **Mol. Weight (Da)** | 22656.47 | 31762.72 | 39856.70 |
| **pI** | 9.88 | 9.32 | 9.76 |
| **Instability Index** | 26.84 (Stable) | 21.19 (Stable) | 35.07 (Stable) |
| **Aliphatic**  **Index** | 68.45 | 84.24 | 77.63 |
| **GRAVY** | -0.878 | -0.528 | -0.811 |
| **Estimated**  **Half -life** | 30 hours (mammalian reticulocytes, in vitro).  >20 hours (yeast, in vivo)  >10 hours (*Escherichia coli*, in vivo). | 30 hours (mammalian reticulocytes, in vitro).  >20 hours (yeast, in vivo)  >10 hours (*Escherichia coli*, in vivo). | 30 hours (mammalian reticulocytes, in vitro).  >20 hours (yeast, in vivo)  >10 hours (*Escherichia coli*, in vivo). |

**Table S11.** Comparative Analysis of Structural Parameters for SM1

| **Model** | **RMSD** | **MolProbity** | **Clash**  **score** | **Poor**  **rotamers** | **Rama**  **favored** |
| --- | --- | --- | --- | --- | --- |
| Initial | 0 | 2.765 | 3.8 | 14.1 | 81.0 |
| MODEL 1 | 0.882 | 1.392 | 1.7 | 0 | 92.4 |
| MODEL 2 | 1.316 | 1.408 | 1.4 | 0 | 90.5 |
| MODEL 3 | 0.710 | 1.264 | 0.8 | 0 | 91.5 |
| MODEL 4 | 0.718 | 1.177 | 0.6 | 0 | 91.9 |
| **MODEL 5** | **0.598** | **1.230** | **0.8** | **0** | **92.4** |

**Table S12.** Comparative Analysis of Structural Parameters for SM2

| **Model** | **RMSD** | **MolProbity** | **Clash**  **score** | **Poor**  **rotamers** | **Rama**  **favored** |
| --- | --- | --- | --- | --- | --- |
| Initial | 0 | 3.279 | 9.7 | 23.3 | 80.3 |
| **MODEL 1** | **0.408** | **2.340** | **21.8** | **0** | **91.3** |
| MODEL 2 | 0.415 | 2.325 | 22.2 | 0.4 | 92.0 |
| MODEL 3 | 0.411 | 2.395 | 19.6 | 1.3 | 91.3 |
| MODEL 4 | 0.395 | 2.514 | 22.0 | 1.8 | 92.0 |
| MODEL 5 | 0.426 | 2.344 | 22.0 | 0.4 | 91.3 |

**Table S13.** Comparative Analysis of Structural Parameters for SM3

| **Model** | **RMSD** | **MolProbity** | **Clash**  **score** | **Poor**  **rotamers** | **Rama**  **favored** |
| --- | --- | --- | --- | --- | --- |
| Initial | 0 | 3.498 | 14.0 | 23.3 | 73.7 |
| MODEL 1 | 0.569 | 2.827 | 26.7 | 2.5 | 87.5 |
| MODEL 2 | 0.557 | 2.766 | 25.4 | 2.2 | 87.0 |
| **MODEL 3** | **0.544** | **2.546** | **27.4** | **1.1** | **88.1** |
| MODEL 4 | 0.554 | 2.735 | 27.2 | 1.8 | 87.0 |
| MODEL 5 | 0.559 | 2.645 | 22.1 | 1.8 | 87.3 |

**Table S14.** Prediction of conformational B-cell epitopes

| **No.** | **Residues** | **Number of**  **residues** | **Score** |
| --- | --- | --- | --- |
| 1 | A:G182, A:D183, A:K184, A:L185, A:V186,  A:A187, A:K188, A:K189, A:K190, A:L192,  A:E194, A:K195, A:A196, A:L197, A:T198,  A:K199, A:K200, A:A201, A:P202, A:P203,  A:H204, A:A205, A:H208 | 23 | 0.794 |

| 2 | A:K102, A:K105, A:E106, A:K107, A:A108,  A:L109, A:G110, A:P111, A:G112 | 9 | 0.769 |
| --- | --- | --- | --- |
| 3 | A:G160, A:A161, A:N162, A:L163, A:D164,  A:K165, A:T166, A:A167, A:Q168, A:K171 | 10 | 0.72 |
| 4 | A:G1, A:I2, A:G3, A:D4, A:P5, A:V6, A:T7, A:C8,  A:L9, A:K10, A:S11, A:G12, A:A13, A:I14,  A:C15, A:H16, A:P17, A:V18, A:F19, A:C20,  A:P21, A:R22, A:Y24, A:K25, A:Q26, A:I27,  A:G28, A:T29, A:C30, A:G31, A:L32, A:P33,  A:G34, A:K36, A:C37, A:K40, A:P41, A:E42,  A:A43, A:A45, A:K46, A:S63, A:Q64, A:A65, A:N66, A:L67, A:G68 | 47 | 0.703 |
| 5 | A:K130, A:T132, A:D133 | 3 | 0.628 |
